# Inhibition of cysteine protease cathepsin Lincreases the level and activity of lysosomal glucocerebrosidase

**DOI:** 10.1172/jci.insight.169594

**Published:** 2024-02-08

**Authors:** Myung Jong Kim, Soojin Kim, Thomas Reinheckel, Dimitri Krainc

**Affiliations:** 1 Department of Neurology, Northwestern University Feinberg School of Medicine, Chicago, Illinois, USA.; 2 Institute of Molecular Medicine and Cell Research, Medical Faculty and BIOSS Centre for Biological Signaling Studies, Albert-Ludwigs-University Freiburg, Freiburg, Germany.

**Keywords:** Cell Biology, Neuroscience, Lysosomes, Parkinson disease

## Abstract

The glucocerebrosidase (GCase) encoded by the GBA1 gene hydrolyzes glucosylceramide (GluCer) to ceramide and glucose in lysosomes. Homozygous or compound heterozygous GBA1 mutations cause the lysosomal storage disease Gaucher disease (GD) due to severe loss of GCase activity. Loss-of-function variants in the GBA1 gene are also the most common genetic risk factor for Parkinson’s disease (PD) and dementia with Lewy bodies (DLB). Restoring lysosomal GCase activity represents an important therapeutic approach for GBA1-associated diseases. We hypothesized that increasing the stability of lysosomal GCase protein could correct deficient GCase activity in these conditions. However, it remains unknown how GCase stability is regulated in the lysosome. We found that cathepsin L, a lysosomal cysteine protease, cleaves GCase and regulates its stability. In support of these data, GCase protein was elevated in the brain of cathepsin L–KO mice. Chemical inhibition of cathepsin L increased both GCase levels and activity in fibroblasts from patients with GD. Importantly, inhibition of cathepsin L in dopaminergic neurons from a patient GBA1-PD led to increased GCase levels and activity as well as reduced phosphorylated α-synuclein. These results suggest that targeting cathepsin L–mediated GCase degradation represents a potential therapeutic strategy for GCase deficiency in PD and related disorders that exhibit decreased GCase activity.

## Abstract 2

<p>Cysteine protease cathepsin L regulates&nbsp;glucocerebrosidase stability and may be a therapeutic target&nbsp;for diseases characterized by&nbsp;glucocerebrosidase deficiency.&nbsp;</p>

## Introduction

Parkinson’s disease (PD) is the second most common neurodegenerative disease and is characterized by multiple motor and nonmotor symptoms. PD causes the progressive loss of dopaminergic neurons in the substantia nigra pars compacta (SNpc). The pathological hallmarks of PD are aggregated, misfolded α-synuclein protein deposits in the form of Lewy bodies and Lewy neurites (1, 2). Several cellular pathways involving impaired mitochondrial quality control, endosomal-lysosomal dysfunction, immune dysfunction, and impaired lysosomal sphingolipid metabolism have been implicated in the pathogenesis of PD (3, 4).

*GBA1* encodes the lysosomal enzyme glucocerebrosidase (GCase), which hydrolyses glucosylceramide (GluCer) to glucose and ceramide in lysosomes. Variations in *GBA1* are considered as one of the most important risk factors for PD, with approximately 5%–10% of patients with PD carrying a *GBA1* variation (5–8). Several studies have found reduced GCase activity in idiopathic PD cases in individuals not carrying *GBA1* variations (9–13), suggesting an important role for GCase activity in the pathogenesis of PD. Clinically, patients with PD with *GBA1* variants show an earlier age at onset, more rapid disease progression, and higher rates of nonmotor symptoms, such as rapid eye movement sleep behavior disorder (RBD) and cognitive impairment, compared with those with non-*GBA1*–associated PD (14–16). Moreover, variants of *GBA1* are also known to increase the risk of dementia with Lewy bodies (DLB) (17–20). Homozygous or compound heterozygous *GBA1* mutations cause the lysosomal storage disease Gaucher disease (GD), due to severe loss of GCase activity (21, 22).

Supporting the strong genetic involvement of the *GBA1* in PD, impaired GCase activity has been shown to cause α-synuclein aggregates in animal and in vitro cell culture models (12, 23–26). Several cellular pathways involving altered sphingolipid profile, autophagy-lysosomal dysfunction, mitochondrial dysfunction, neuroinflammation or endoplasmic reticulum (ER) stress have been suggested as pathogenic mechanisms of *GBA1*-PD (12, 27–33). Most of PD-associated *GBA1* variations lead to a reduction in GCase activity in lysosomes, due to impaired GCase transport from the ER to the lysosome, reduced intrinsic enzymatic activity of the GCase, or a haplo-insufficiency of *GBA1* gene (34–36). Thus, restoring the reduced GCase activity in the lysosome is crucial to combatting *GBA1*-associated diseases, and multiple therapeutic approaches have been developed. Misfolded GCase proteins with pathogenic genetic variations are retained in the ER and undergo ER-associated degradation (ERAD), resulting in a reduction in GCase protein levels in lysosomes (36, 37). Ambroxol, a well-known expectorant, has been reported to facilitate lysosomal transport of misfolded GCase proteins by acting as a chemical chaperone of the misfolded GCase protein in the ER. Ambroxol has been reported to increase both the lysosomal fraction and the enzymatic activity of GCase variants in fibroblasts from patients with GD (38, 39). In addition to chemical chaperoning of the misfolded GCase protein, other therapeutic strategies such as adeno-associated virus–mediated (AAV-mediated) recombinant WT GCase transport or chemical GCase activators have recently been developed (40–43). Since each individual therapeutic strategy has specific strengths and limitations, it will be important to have multiple therapeutic strategies to combat the disease. Here, we present a potentially novel therapeutic strategy that targets GCase stability in lysosomes. Conceptually, inhibition of GCase degradation in lysosomes should increase GCase protein abundance and activity in lysosomes. However, it is still unknown which proteases are directly involved in GCase degradation in lysosomes. In this study, we show that lysosomal cysteine protease cathepsin L regulated GCase stability and suggest that chemical inhibition of cathepsin L activity represents a potential therapeutic strategy for *GBA1*-associated PD and related diseases.

## Results

### Genetic inhibition of cathepsin L increases GCase protein level.

**Figure 1:**
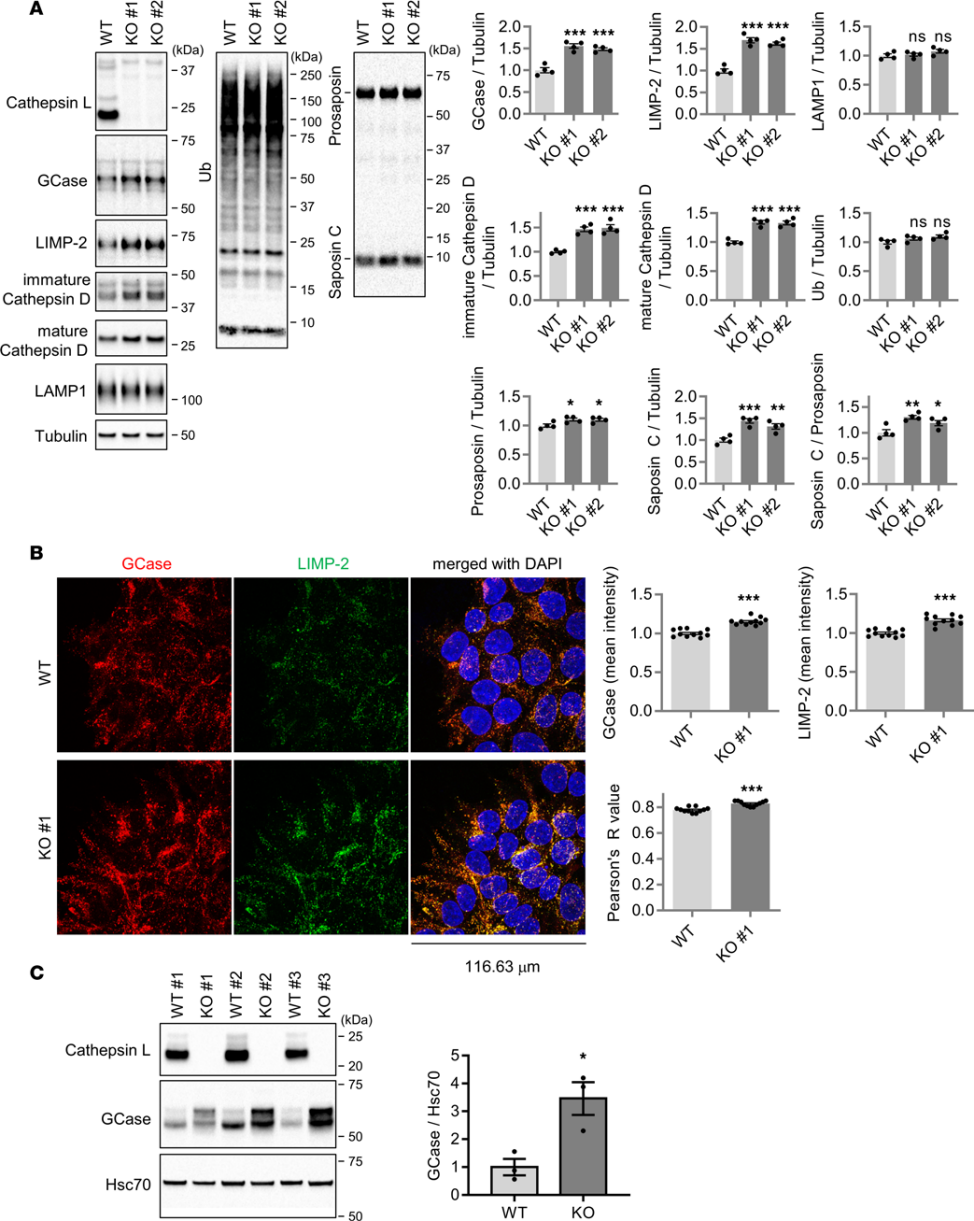
GCase protein levels are increased in cathepsin L–KO cells. (**A**) Representative immunoblot data and quantification of indicated proteins in WT and cathepsin L–KO cell lines. Band intensities were normalized to tubulin and compared with the WT cells. Data are mean ± SEM. One-way ANOVA followed by Dunnett’s test. *n* = 4. *P < 0.05; **P < 0.01; ***P < 0.001. (**B**) Representative immunofluorescence image of GCase and LIMP-2 in WT and cathepsin L–KO cells. Quantitation of mean fluorescence intensity and Pearson’s correlation coefficient of GCase and LIMP-2 are shown. *n* = 11 microscopic fields from 2 coverslips for WT, *n* = 11 microscopic fields from 2 coverslips for KO cells. ***P < 0.001; 2-tailed unpaired *t* test. (**C**) Analysis of GCase levels in brain lysates from cathepsin L–deficient mice. Western blot analysis of cortical lysates from 5-month-old WT and *Ctsl^−/−^* mice with indicated antibodies. Data presented as mean ± SEM. *n* = 3. Two-tailed Student’s *t* test; *P < 0.05. GCase protein levels were normalized with Hsc70 protein level.

Lysosomal cysteine proteases are critical for protein turnover in the endolysosomal cell compartment (44–46). In particular, cathepsin L has been previously implicated in the proteolytic processing of progranulin, suggesting its potential role in the pathogenesis of frontotemporal dementia (47). To explore potentially novel cellular functions of cathepsin L, we generated 4 independent cathepsin L–KO human embryonic kidney 293-FT (HEK293-FT) cell lines by using CRISPR/Cas9 gene editing. Immunoblot analysis using cathepsin L antibody confirmed complete loss of cathepsin L protein in 4 cathepsin L–KO cell lines (Figure 1A and Supplemental Figure 1A; supplemental material available online with this article; https://doi.org/10.1172/jci.insight.169594DS1). Consistent with the previous report demonstrating that cathepsin L degrades lysosomal protease cathepsin D in cells (48), both mature and immature cathepsin D protein levels were elevated in 4 independent cathepsin L–KO cell lines (Figure 1A and Supplemental Figure 1A). Notably, we found that GCase protein levels were significantly elevated in the 4 cathepsin L–KO cell lines, compared with the WT cells (Figure 1A and Supplemental Figure 1A). While the levels of lysosomal associated membrane protein 1 (LAMP1) were not changed, the levels of LIMP-2, a receptor for lysosomal transport of GCase (49), were increased in cathepsin L–KO lines (Figure 1A and Supplemental Figure 1A). Lysosomal GCase activity is regulated by saposin C that acts as a physiological activator of GCase (50–53). We found that Saposin C levels and the ratio of Saposin C to prosaposin were increased in cathepsin L–KO cells (Figure 1A and Supplemental Figure 1A), suggesting that cathepsin L may be involved in the degradation of saposin C in lysosomes.

Supporting the immunoblot data, immunofluorescence staining of GCase and LIMP-2 confirmed that both GCase and LIMP-2 protein levels were increased in the cathepsin L–KO cells (Figure 1B). The colocalization of GCase and LIMP-2 was also significantly increased in the cathepsin L–KO cells (KO#1), compared with the WT cells (Figure 1B). The immunostaining of GCase and LIMP-2 proteins in 2 additional cathepsin L–KO cell lines (KO#3 and KO#4) also showed similar results (Supplemental Figure 2). Unlike canonical sorting receptors that cycle between the Golgi and lysosomes, LIMP-2 resides mainly in the lysosomes (49), suggesting that higher colocalization of GCase with LIMP-2 was due to increased GCase abundances in the lysosome. Consistent with the data from the CRISPR cathepsin L–KO cell lines, the transient knockdown of endogenous cathepsin L with 2 independent RNAi constructs also increased the levels of GCase, LIMP-2, and cathepsin D (Supplemental Figure 3). To confirm the finding in vivo, we found that GCase protein levels were significantly increased in the cathepsin L–KO mouse brain lysates, compared with WT mouse lysates (Figure 1C), further indicating that cathepsin L regulates GCase levels.

### Lysosomal GCase activity is increased in the cathepsin L–KO cells.

**Figure 2:**
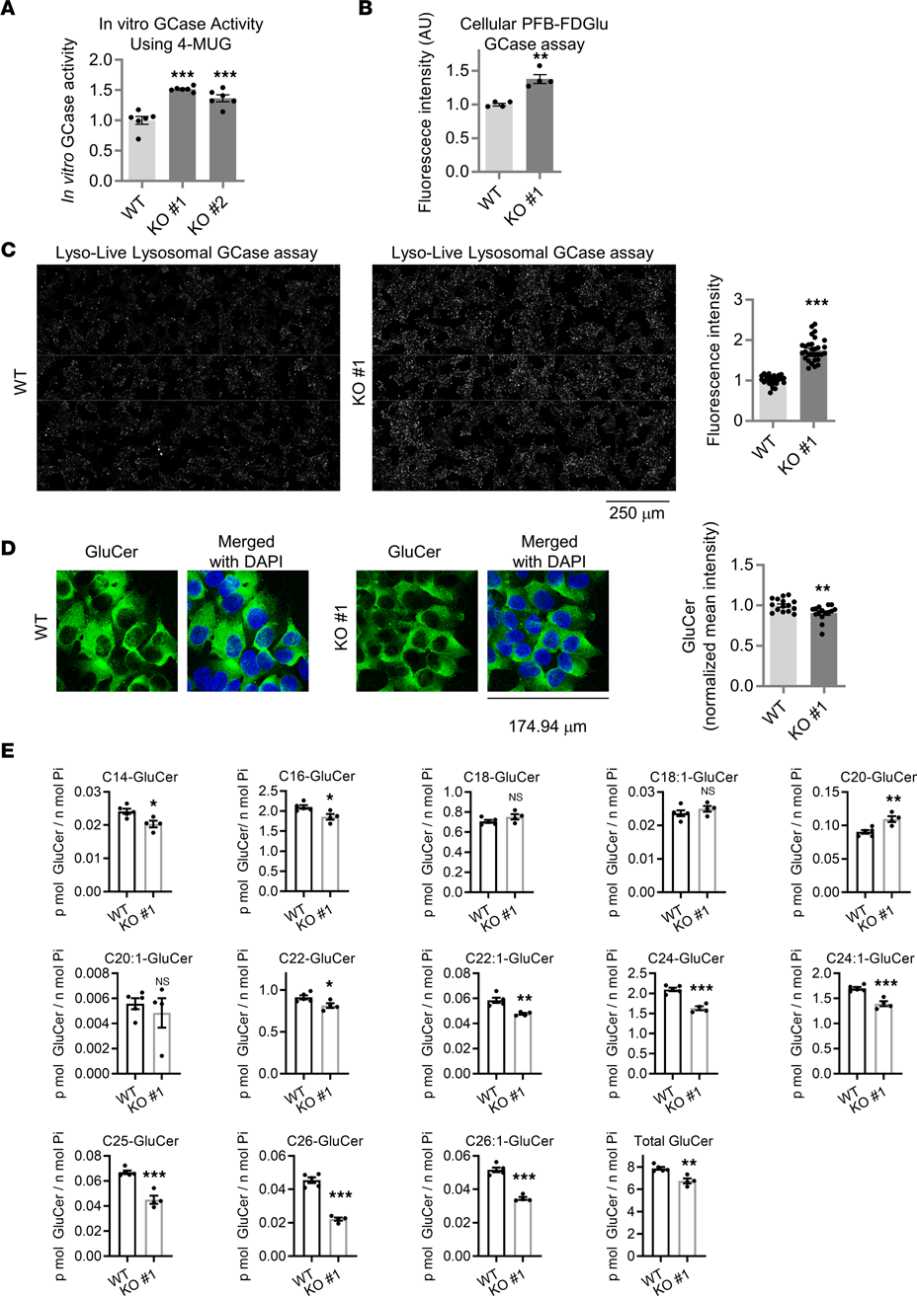
Lysosomal GCase activity is increased and GluCer levels are reduced in cathepsin L–KO cells. (**A**) In vitro GCase activity in WT HEK293-FT and cathepsin L–KO cell lysates. Lysates from WT or cathepsin L–KO cells were assayed for in vitro GCase assay using 4-MUG substrates. Data are mean ± SEM. One-way ANOVA followed by Dunnett’s test; ***P < 0.001. *n* = 6. (**B**) PFB-FDGlu GCase activity of WT HEK293-FT and cathepsin L–KO cells. Live WT HEK293-FT and cathepsin L–KO cells were labeled with 100 μM PFB-Glu for 2 hours in OPTI-MEM media. After washing with PBS twice, cells were lysed with RIPA buffer and fluorescence intensities were measured. Data normalized with protein concentration of cell lysates and compared with the WT. *n* = 4 for WT, *n* = 4 for KO cells. **P < 0.01; 2-tailed unpaired *t* test. (**C**) LysoLive-GCase activity of WT HEK293-FT and cathepsin L–KO cells. Representative fluorescence images from LysoLive-GCase assay are shown. Graph shows mean fluorescence intensity from the LysoLive-GCase assay. Data were compared with the WT cells. *n* = 27 microscopic fields from 3 independent dishes for WT, 30 microscopic fields from 3 independent dishes for cathepsin L–KO cells. Two-tailed unpaired *t* test, ***P < 0.001. Scale bar: 250 μm. (**D**) GluCer staining of WT HEK293-FT and cathepsin L–KO cells. Cells were stained with anti-GluCer antibody. Graph shows mean fluorescence intensity of GluCer staining. *n* = 15 microscopic fields from 2 coverslips for WT, *n* = 15 microscopic fields from 2 coverslips for cathepsin L–KO cells. Two-tailed unpaired *t* test; **P < 0.01. (**E**) Lipidomic analysis of GluCer species in WT HEK293-FT cells and cathepsin L–KO#1 cells. GluCer were quantified and expressed as p mol/n mole inorganic phosphate (Pi). Two-tailed unpaired *t* test; *P < 0.05, **P < 0.01, ***P < 0.001. *n* = 5 for WT and *n* = 4 for KO#1.

To determine whether the increased GCase protein level in the cathepsin L–KO cells resulted in an increased GCase activity, we first performed in vitro GCase activity assay by using cell lysates and 4-methylumbelliferyl-β-D-glucopyranoside (4-MUG), an artificial fluorometric GCase substrate. As shown in Figure 2A and Supplemental Figure 1B, the lysates of cathepsin L–KO cells showed higher in vitro GCase activity, compared with the WT cell lysates. However, it is worth pointing out that this in vitro GCase assay using extracted cell lysates and 4-MUG substrate does not account for proper lysosomal targeting of GCase, lysosomal pH, or the presence of cofactor that could influence GCase activity in living cells. To address this concern, we used 2 independent cell penetrant artificial GCase substrates to assay GCase activity in living cells. Using 5-(Pentafluorobenzoylamino) Fluorescein Di-β-D-Glucopyranoside (PFB-FDGlu), a fluorescent quenched probe that yields fluorescein fluorescence upon hydrolysis by GCase (54), we show that GCase activity was significantly increased in the cathepsin L–KO cells, compared with the WT cells (Figure 2B). In order to examine lysosomal GCase activity, we visualized fluorescent cleavage product of lysosome-targeted GCase substrate (GlucGreen), whereby lysosome-targeted GCase substrates become fluorescent only upon GCase enzymatic activity (55). Using this assay, we found that cathepsin L–KO cells displayed a significant increase in fluorescence intensity, compared with the WT cells (Figure 2C). Next, we tested whether GluCer levels were altered in the cathepsin L–KO cells, since GluCer is the primary lipid substrate of GCase enzyme. Indeed, immunofluorescence staining using an anti-GluCer antibody showed that GluCer levels are significantly reduced in the cathepsin L–KO cells, compared with the WT cells (Figure 2D). The lipidomic investigation using mass spectrometry (MS) revealed a significant reduction in the total GluCer levels in the cathepsin L–KO cell line. Notably, GluCer species characterized by very long acyl chains (such as C24, C24:1, C25, C26, and C26:1) showed a more consistent and pronounced decrease in the cathepsin L–KO cell line, while GluCer species with C16 and C22 acyl chains exhibited a modest decrease (Figure 2E).

### Cathepsin L cleaves GCase in vitro, and regulates GCase stability in cells.

**Figure 3:**
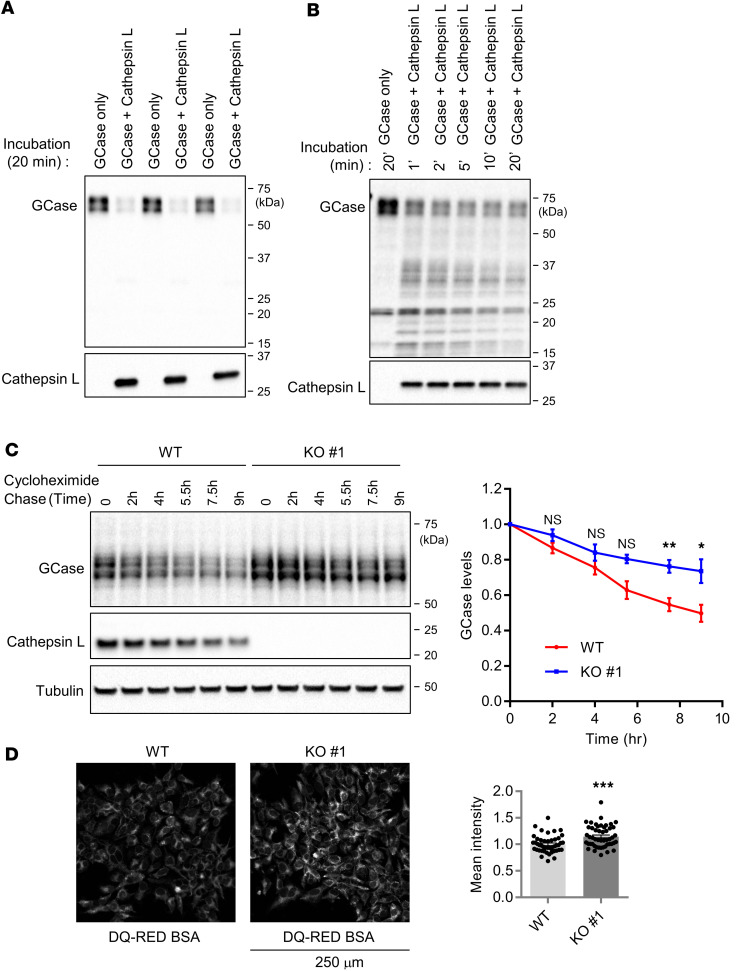
GCase protein stability and lysosomal proteolysis in cathepsin L–KO cells. (**A**) In vitro cleavage assay using recombinant cathepsin L and GCase. Recombinant GCase and cathepsin L were incubated as indicated. After 20 minutes of incubation at room temperature, protein samples were immunoblotted with antibodies against GCase and cathepsin L. (**B**) Time-course study of cathepsin L–mediated GCase cleavage in vitro. Recombinant GCase and cathepsin L incubated for indicated times. Protein samples were immunoblotted with antibodies against GCase and cathepsin L. (**C**) GCase stabilities in WT and cathepsin L–KO cells. WT and cathepsin L–KO cells were treated with 100 μM cycloheximide for indicated times. Cell lysates were immunoblotted with antibodies against GCase, cathepsin L, and tubulin. Band intensities were normalized with tubulin and compared with 0-hour samples. Data represent mean ± SEM. *n* = 3 independent experiments. Paired 2-tailed Student’s *t* test. *P < 0.05, **P < 0.01, compare with the same time point of WT cells. (**D**) Representative images of DQ-RED BSA proteolysis assay. WT and cathepsin L–KO cells were labeled with 10 µg/ mL DQ-RED BSA for 4 hours in OPTI-MEM. Graph shows as mean fluorescence intensity of cleaved DQ-RED BSA ± SEM. *n* = 50 microscopic fields from 4 dishes for WT and *n* = 53 microscopic fields from 4 dishes for cathepsin L–KO cells. Scale bar: 250 μm. Unpaired 2-tailed Student’s *t* test; ***P < 0.01.

To determine whether GCase can be cleaved by cathepsin L, we first performed in vitro cleavage assay using purified recombinant cathepsin L and GCase proteins. The in vitro cleavage assay showed that GCase proteins are readily degraded by the coincubation with recombinant cathepsin L (Figure 3A). The time-course experiment of in vitro cleavage assay showed that cathepsin L cleaves most of GCase proteins within a minute (Figure 3B). Furthermore, to monitor GCase stability in cells, we utilized cycloheximide, an inhibitor for protein translation (56). Compared with the WT cells, the cathepsin L–KO cells showed a slowed GCase reduction in the presence of cycloheximide, indicating that GCase stability is increased by the loss of cathepsin L in cells (Figure 3C). The increased GCase stability could be due to potentially altered lysosomal proteolysis activity of the cathepsin L–KO cells, since cathepsin L is one of the many lysosomal proteases. To address this, we measured total lysosomal proteolysis by DQ-Red BSA protease activity assay (57). This technique relies on cleavage of the self-quenched DQ-Red BSA protease substrates in lysosomes and other acidic compartments to generate a highly fluorescent product. Using this approach, we found that the proteolysis activity was not impaired in the cathepsin L–KO cells, compared with the WT cells (Figure 3D). Similar to our results, a recent study also found that cathepsin L loss in mouse embryonic fibroblast (MEF) cells impaired neither DQ-BSA degradation nor general protein catabolism (58–60). Considering that cathepsin D, a previously known cathepsin L substrate, is significantly increased in the cathepsin L–KO cells (Figure 1A), the increased DQ-Red BSA protease activity could be due to potential compensatory upregulation of other lysosomal proteases, including cathepsin D.

As an independent strategy to assess lysosomal degradation capacity, we monitored the clearance rate of endogenous cellular proteins upon nutrient starvation that leads to ubiquitination of intracellular proteins and encapsulation into autophagosomes. The ubiquitinated cellular proteins in autophagosomes are delivered to lysosomes and degraded by lysosomal proteases (61). Despite the complete loss of cathepsin L, the clearance rates of both ubiquitinated intracellular proteins and LC3B, an autophagy adaptor, induced by nutrient starvation were not significantly different between the cathepsin L–KO and WT cells (Supplemental Figure 4), suggesting that the loss of cathepsin L did not affect the lysosomal degradation capacity. Thus, these data suggest that the increased GCase stability in the cathepsin L–KO cells is not due to defects in total lysosomal degradation activity, but rather that GCase abundance is directly regulated by cathepsin L.

### Chemical inhibition of cathepsin L increases GCase level, in vitro GCase activity, and live cell GCase activity in microglial cells and patient-derived fibroblasts.

**Figure 4:**
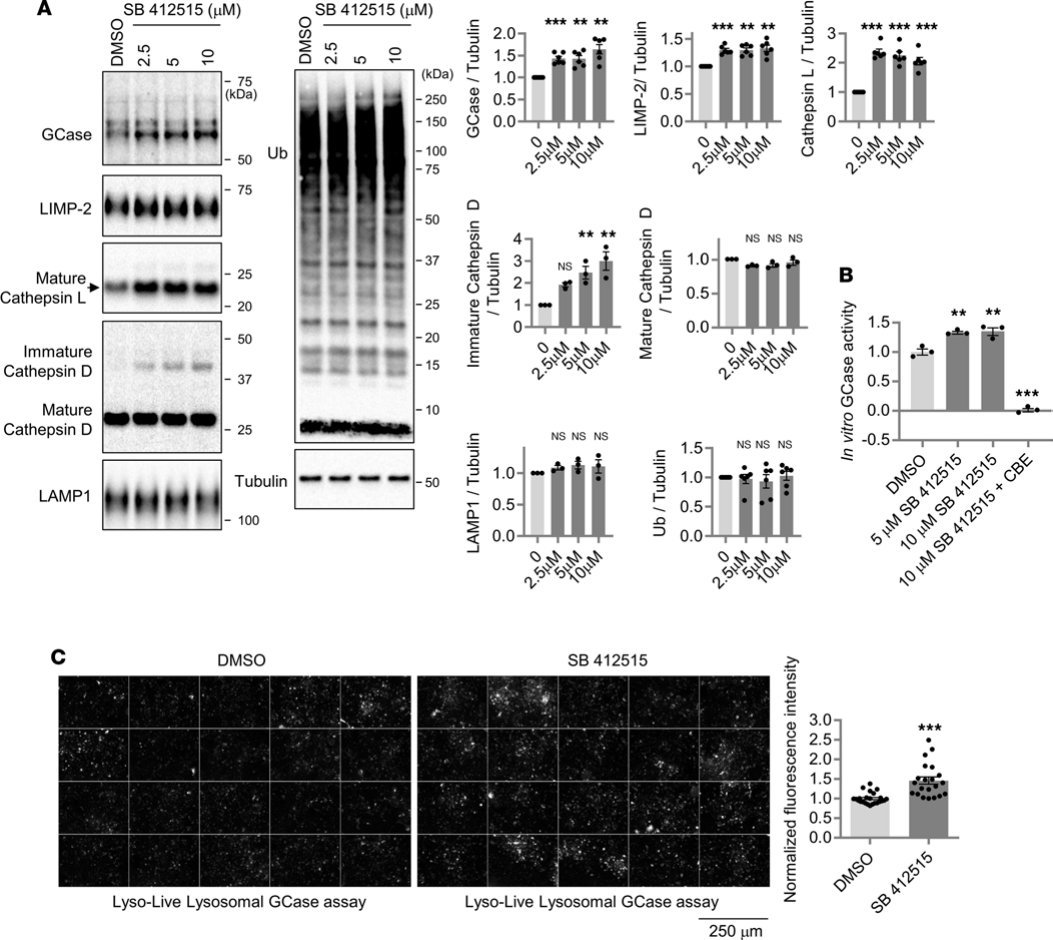
Chemical inhibition of cathepsin L increases both GCase protein level and GCase activity in microglial cells. (**A**) Effects of SB 412515, a reversible cathepsin L inhibitor, on GCase level in HMC3 cells. Human microglial HMC3 cells were treated with either DMSO or SB 412515 (2.5, 5, and 10 μM) for about 20 hours. Cell lysates were immunoblotted with indicated antibodies. Band intensities are normalized with tubulin levels and compared with the DMSO-treated HMC3 cells. Data are mean ± SEM. One-way ANOVA followed by Dunnett’s test; ***P < 0.001; **P < 0.01. *n* = 6. (**B**) Effects of cathepsin L inhibitor (SB 412515) on in vitro GCase activity in HMC3 cells. HMC3 cells were treated with either DMSO or SB 412515 (5 μM and 10 μM) for about 20 hours. In total, 10 μg of cleared cell lysates were subjected to in vitro GCase assay. *CBE* is an irreversible inhibitor of GCase. Data are mean ± SEM. One-way ANOVA followed by Dunnett’s test; ***P < 0.001; **P < 0.01. *n* = 3. (**C**) Effects of cathepsin L inhibitor (SB 412515, 5 μM, ~20 hours) on LysoLive-GCase activity in HMC3 cells. Representative fluorescence images from LysoLive-GCase activity assay are shown. Scale bar: 250 μm. Graph shows mean fluorescence intensity from the LysoLive-GCase activity. Data were compared with the DMSO-treated HMC3 cells. Two-tailed unpaired *t* test; ***P < 0.001. *n* = 24 microscopic fields from 3 dishes for the DMSO-treated cells, and *n* = 22 microscopic fields from 3 dishes for the SB 412515-treated cells.

It has been reported that the expression levels of both cathepsin L and GCase are higher in microglia than in other cells of the brain (62). It is conceivable that microglial cells may be affected by GCase deficiency, since macrophages are severely affected in patients with GD (21, 63). Although GCase function in microglia has not been studied at a molecular level, several studies suggest that GCase deficiency causes microglial dysfunction (64–68). Hence, we asked whether the cathepsin L–mediated GCase regulation also operates in microglia. As a model system, we utilized the Human Microglia Clone 3 (HMC3) cells, an immortalized human microglia cell line derived from human fetal brain primary microglia culture (69). The HMC3 cell is known to express IBA1, a microglia/macrophage-specific calcium binding protein, and the mRNA expression of microglia-lineage markers (P2RY12 and CSF1R) in HMC3 cells has been previously reported (69). In vitro GCase activity and GCase protein levels in HMC3 cells were approximately a third of those observed in HEK293-FT cells (Supplemental Figure 5). We used a chemical inhibitor of cathepsin L to test whether cathepsin L activity regulates GCase abundance in the microglial HMC3 cells. To avoid potential covalent irreversible inhibition of other related lysosomal cysteine family proteases, we used a reversible cathepsin L inhibitor (SB 412515, a reversible peptidyl aldehyde inhibitor of cathepsin L) (70). SB 412515 exhibits a 30-fold preference for inhibiting cathepsin L over cathepsin K (71). We found that the treatment of the microglial HMC3 cells with SB 412515 increased both GCase and LIMP-2 levels (Figure 4A), whereas neither LAMP1 nor ubiquitinated protein levels were changed (Figure 4A). Consistent with our data that genetic KO of the cathepsin L gene resulted in a significant increase in both immature and mature forms of cathepsin D (Figure 1A and Supplemental Figure 1A), treatment with SB 412515 led to a significant increase in the levels of immature forms of cathepsin D, whereas the levels of mature forms of cathepsin D remained unchanged (Figure 4A). Of note, SB 412515 also increased mature cathepsin L levels (Figure 4A), presumably due to the stabilization of inactive cathepsin L bound to the cathepsin L inhibitor. Supporting the data that the SB 412515 treatment increased GCase protein level, we also found increased GCase activity in HMC3 cell lysates (Figure 4B). The majority of this GCase activity was abolished by *CBE*, an irreversible inhibitor of GCase (Figure 4B). When we also analyzed live cell GCase activity using the LysoLive GCase activity assay, the HMC3 cells treated with SB 412515 displayed a significant increase in fluorescence intensity (Figure 4C), indicating that lysosomal GCase activity was increased by the chemical inhibition of cathepsin L in microglial cells.

A recent single-nucleus RNA-Seq study using a neuronopathic GD (nGD) mouse brain (64) revealed a significant increase in cathepsin L mRNA levels specifically within the microglia of nGD mouse brains. To further explore the impact of *GBA1* deficiency on cathepsin L protein levels, we established a *GBA1*-KO human microglial cell line 3 (HMC3) and utilized a previously characterized GBA1-KO HEK293-FT cell line (28). Biochemical analysis of these *GBA1*-KO cell lines revealed significant increases in the mature form of cathepsin L, in comparison with their respective parental WT controls (Supplemental Figure 6 for HMC3 cell line and Supplemental Figure 7 for HEK293-FT cell line). Interestingly, no notable changes were observed in the levels of the immature form of cathepsin L. Additionally, both the *GBA1*-KO HMC3 and *GBA1*-KO HEK293-FT cell lines exhibited a substantial decrease in the levels of mature cathepsin D when compared with their parental WT controls (Supplemental Figure 6 for HMC3 cell line and Supplemental Figure 7 for HEK293-FT cell line). This finding aligns with previous research suggesting cathepsin L’s involvement in cathepsin D degradation within lysosomes (48).

**Figure 5:**
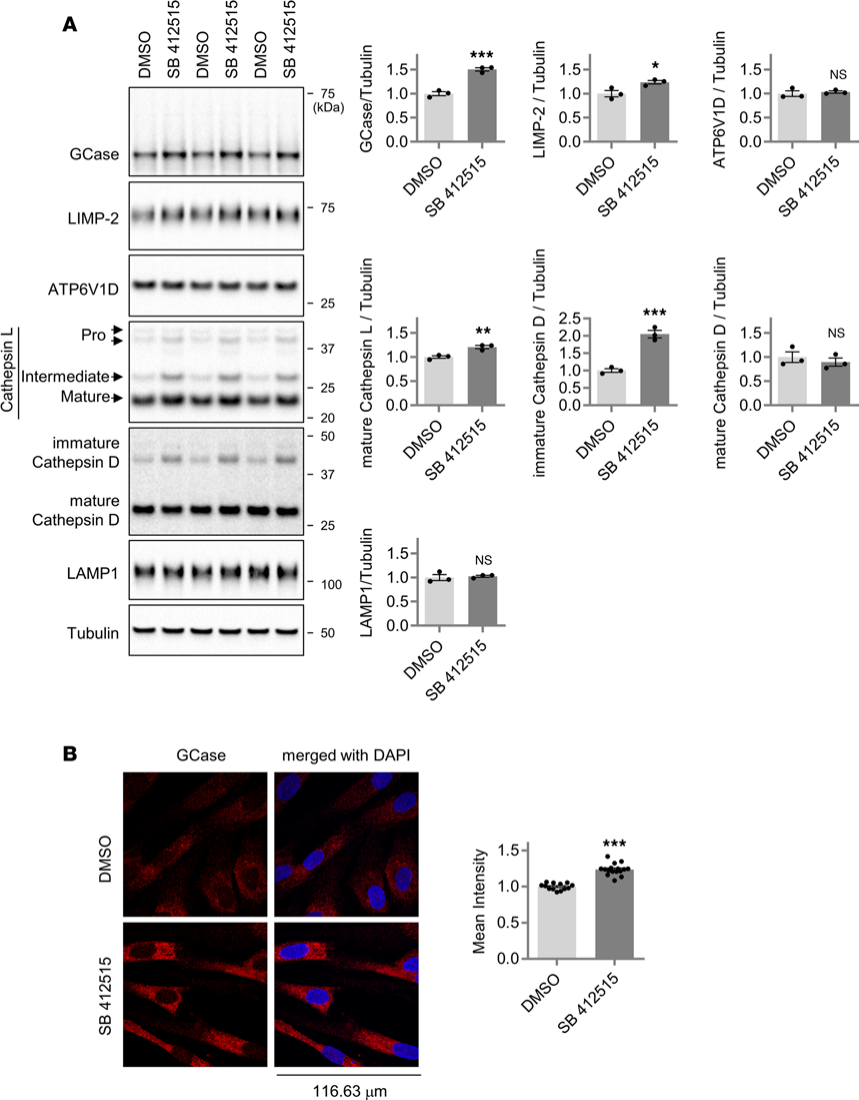
Chemical inhibition of cathepsin L increases GCase protein level in GD patient fibroblasts (*GBA1* L44P/L444P). (**A**) Effect of cathepsin L inhibitor (SB 412515) on GCase protein levels in GD fibroblast cells. GD fibroblast cells were treated with either DMSO or SB 412515 (5 μM) for about 20 hours. Cell lysates were immunoblotted with antibodies against GCase, LIMP-2, ATP6V1D, cathepsin L, cathepsin D, LAMP1, and tubulin. Band intensities were normalized to tubulin and compared with the DMSO-treated cells. Two-tailed unpaired *t* test; *P < 0.05; **P < 0.01; ***P < 0.001. *n* = 3. (**B**) Representative fluorescence image of GCase staining in GD fibroblast cells. GD fibroblast cells were treated with either DMSO or 7.5 μM SB 412515 for about 20 hours. GD fibroblast cells were then immunostained for GCase. Scale bar: 116.63 μm. Data presented as mean GCase fluorescence intensity ± SEM. *n* = 13 microscopic fields from 2 coverslips for DMSO-treated cells, and *n* = 16 microscopic fields from 2 coverslips for cathepsin L inhibitor–treated cells. Two-tailed unpaired *t* test; ***P < 0.001.

**Figure 6:**
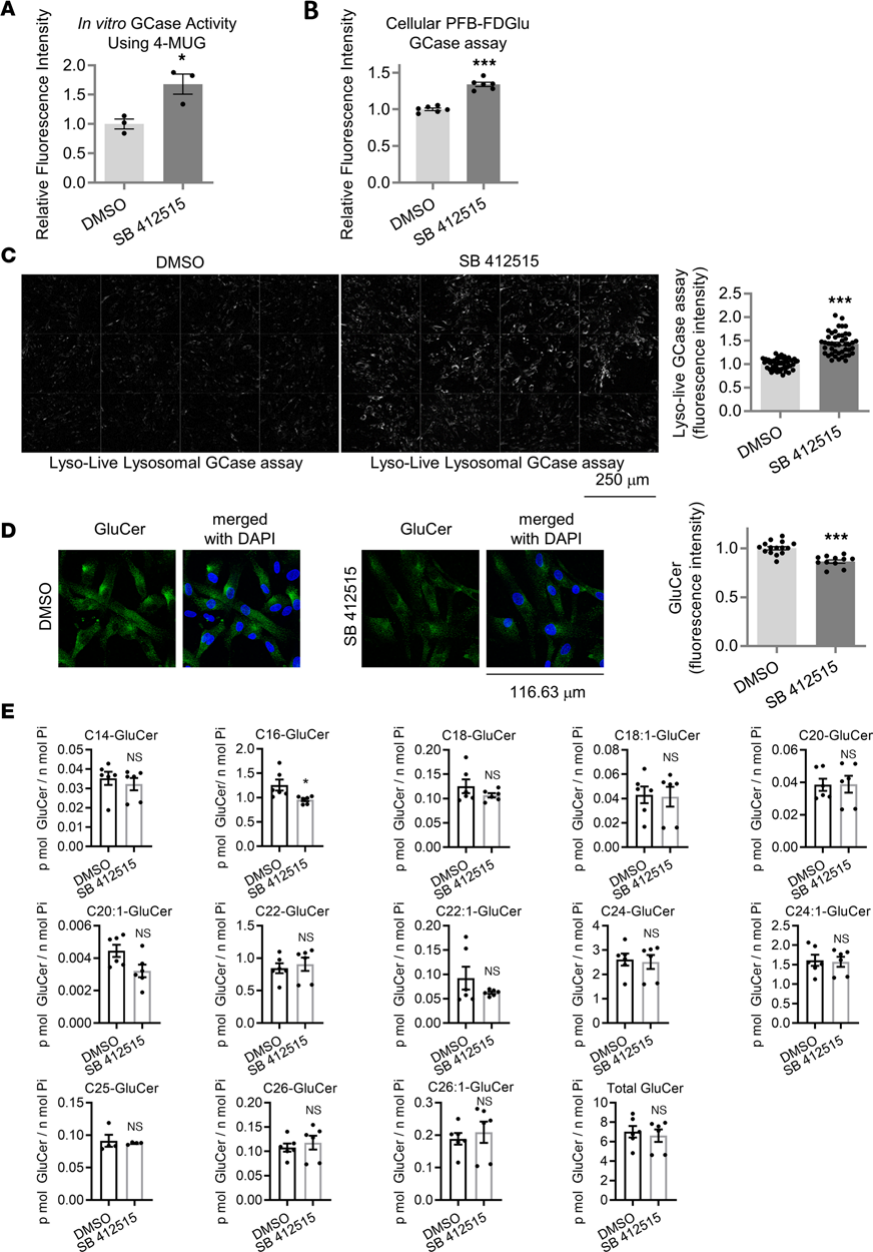
Inhibitor of Cathepsin L increases GCase activity and GluCer levels in GD human fibroblast cells (GBA1 L444P/L444P). (**A**) GCase activity in GD fibroblasts treated with either DMSO or 5 μM SB 412515 for ~20 hours (hrs). Data are normalized mean ± SEM. Two-tailed unpaired *t* test, *P < 0.05, *n* = 3 per condition. (**B**) Quantification of PFB-FDGlu GCase activity assay. Live GD fibroblast cells were treated with either DMSO or 5 μM SB 412515 for ~20 hrs. Graph shows mean fluorescence intensity from the PFB-FDGlu GCase assay. Data were normalized with the protein concentration of cell lysates. *n* = 6 per condition, ***P < 0.001, 2-tailed unpaired *t* test. (**C**) Cathepsin L inhibitor (SB 412515, 5 μM, ~20 hrs) effects on live lysosomal GCase activity in the GD fibroblasts. Representative images of LysoLive-GCase activity and mean fluorescence intensity is shown. Data were compared with the DMSO-treated WT cells. Unpaired 2-tailed *t* test, ***P < 0.001. *n* = 46 fields from 3 dishes for the DMSO-treated cells, and *n* = 45 fields from 3 dishes for the SB 412515-treated cells. Scale bar: 250 µm. (**D**) GluCer staining in the L444P GD fibroblast cells upon cathepsin L inhibitor treatment (SB 412515, 5 μM, ~20 hrs). Representative images and mean fluorescence intensity is shown. Data were compared with the DMSO-treated WT cells. *n* = 15 fields from 2 coverslips for the DMSO-treated cells, and *n* = 11 fields from 2 coverslips for the SB 412515-treated cells. Unpaired 2-tailed t test, ***P < 0.001. Scale bar: 116.63 µm. (**E**) Lipidomic analysis of GluCer species upon cathepsin L inhibitor treatment in L444P fibroblast cells treated either DMSO or 5 μM of SB 412515 for ~20 hrs. GluCer species were compared with DMSO-treated samples. *P < 0.05, 2-tailed unpaired *t* test. *n* = 6, except for C25-GluCer (*n* = 4).

Having found that the treatment of cathepsin L inhibitor in the HMC3 cells increased both GCase protein level and lysosomal GCase activity, we asked whether the cathepsin L inhibitor can correct reduced GCase protein level and GCase activity in cellular models of GCase deficiency, such as human fibroblast cells derived from a patient with GD carrying a homozygous L444P mutation (*GBA1* L444P/L444P) (72). Indeed, the treatment of GD fibroblast cells with SB 412515 increased both GCase and LIMP-2 levels (Figure 5A). The levels of pro at lower band (not quantified), intermediate, and mature forms of cathepsin L were also increased by SB 412515 treatment (Figure 5A). The levels of lysosomal proton pump subunit ATP6V1D, mature cathepsin D, and LAMP1 remained unchanged following SB 412515 treatment, whereas the levels of immature cathepsin D were increased (Figure 5A). Supporting these data, immunofluorescence staining using GCase antibody or LIMP-2 antibody also showed that both GCase (Figure 5B) and LIMP-2 protein levels (Supplemental Figure 8), as well as GCase activity, were increased by the SB 412515 treatment of GD fibroblasts (Figure 6A). Moreover, both PFB-FDGlu GCase assay (Figure 6B) and LysoLive GCase assay (Figure 6C) showed that the SB 412515 treatment in GD fibroblast cells increased lysosomal GCase activity in living cells. In addition, we found that GluCer levels in GD fibroblasts were significantly reduced by the SB 412515 treatment, as assayed with immunofluorescence staining using an anti-GluCer antibody (Figure 6D). To further examine the effects of SB 412515 in L444P fibroblast cells, we conducted a MS-based lipid analysis (Figure 6E). The lipidomic study revealed that the L444P fibroblast cell line contained 4 major GluCer species (C16-GluCer, C22-GluCer, C24-GluCer, and C24:1-GluCer). Treating the L444P fibroblast cells with SB 412515 for a day did not result in a significant reduction in total GluCer levels. However, upon analyzing individual GluCer species, we found that C16-GluCer exhibited a notable 24% reduction with SB 412515 treatment, whereas C22, C24, and C24:1-GluCer did not exhibit statistically significant changes (Figure 6E). These findings suggest that C16-GluCer may have a shorter half-life in cells compared with other GluCer species with longer acyl chains.

### Chemical inhibition of cathepsin L increases GCase levels and reduces phosphoSer-129-α-synuclein (pS129-α-synuclein) in GBA1-PD dopaminergic neurons.

**Figure 7:**
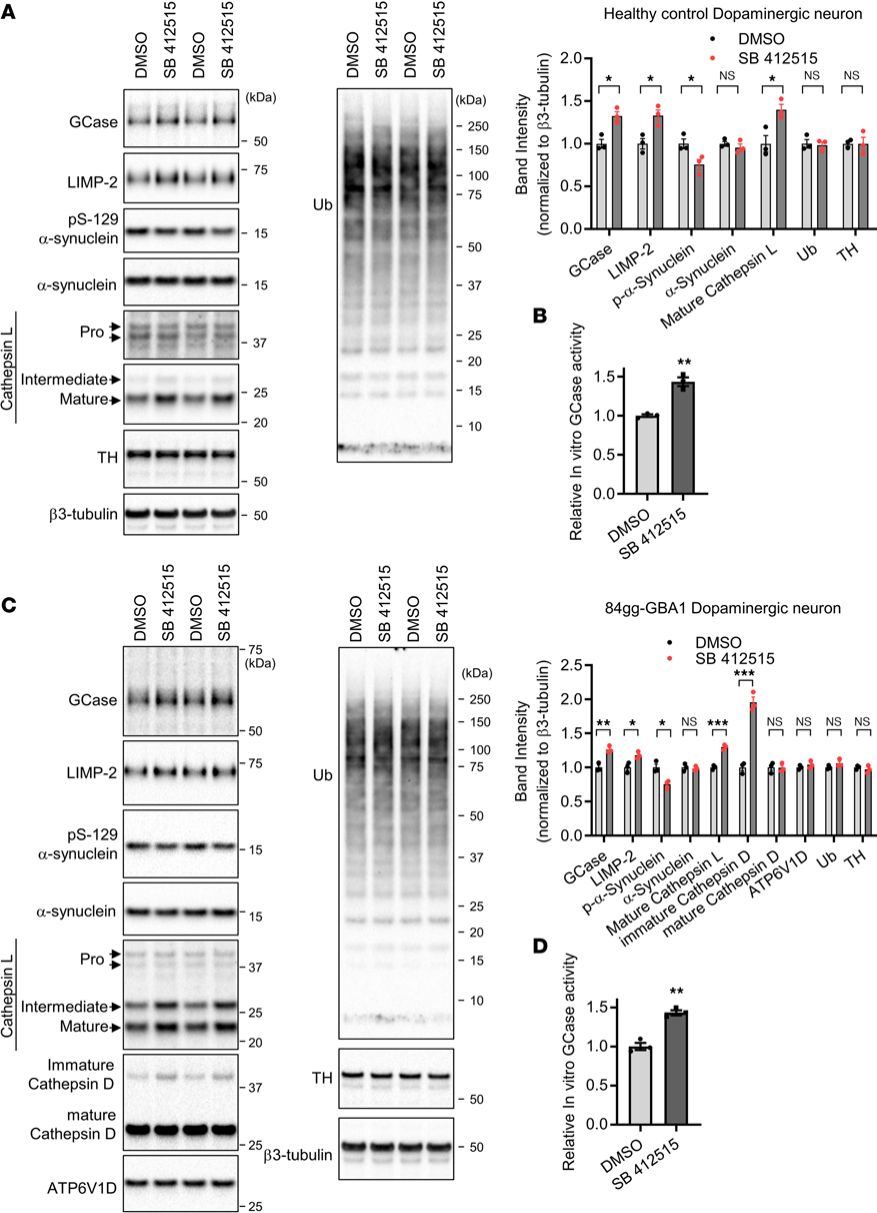
Chemical inhibition of cathepsin L increases GCase levels and reduces pS129-α-synuclein in dopaminergic neurons derived from a patient with GBA1-PD. (**A**) Control iPSC-dopaminergic neurons derived from a healthy individual at DIV 46 were treated with either DMSO or 5 μM of cathepsin L inhibitor SB 412515 every 2 days for 4 days. Band intensities were normalized to β3-tubulin. The data are presented as the mean ± SEM, *n* = 3; *P < 0.05, using unpaired 2-tailed *t* test. (**B**) Cathepsin L inhibitor (SB 412515) effects on in vitro GCase activity in healthy control iPSC-dopaminergic neurons. Control neurons derived from a healthy individual at DIV 46 were treated with either DMSO or 5 μM SB 412515 every 2 days for 4 days. GCase activity was assayed using 4-MUG substrates. Data are mean ± SEM. Two-tailed unpaired *t* test. **P < 0.01, *n* = 3. (**C**) Cathepsin L inhibitor (SB 412515) effects on GCase and pS129-α-synuclein levels in mutant GBA1 (heterozygous GBA1-c.84dupG frameshift) neurons derived from a patient with PD. Neurons were treated with either DMSO or 5 μM SB 412515 every 2 days for 4 days. Cell lysates were analyzed with immunoblot using indicated antibodies. Band intensities were normalized to β3-tubulin and compared with the DMSO-treated cells. The data are presented as the mean ± SEM, *n* = 3; *P < 0.05, **P < 0.01, ***P < 0.001, using unpaired 2-tailed *t* test. (**D**) Cathepsin L inhibitor (SB 412515) on GCase activity in mutant GBA1 (heterozygous GBA1-c.84dupG frameshift) neurons. Neurons were treated with either DMSO or 5 μM SB 412515 every 2 days for 4 days. In total, 10 µg of lysates were assayed for in vitro GCase assay using 4-MUG substrates. Data are mean ± SEM. Two-tailed unpaired *t* test. **P < 0.01, *n* = 3.

Since our data show that cathepsin L regulates GCase abundance in nonneuronal cells, we next examined whether cathepsin L regulates GCase abundance in dopaminergic neurons derived from a patient with *GBA1*-PD. Similar to the data obtained from microglia cell line and GD fibroblasts, treatment with SB 412515 in the control induced pluripotent stem cell–dopaminergic (iPSC-dopaminergic) neurons derived from a healthy individual elevated both GCase and LIMP-2 protein levels (Figure 7A). Moreover, the level of pS129-α-synuclein, a modified form of α-synuclein that is associated with PD pathogenesis (73), was significantly reduced by the SB 412515 treatment, whereas the total α-synuclein levels were not significantly changed. Importantly, SB 412515did not significantly alter the levels of tyrosine hydroxylase (TH), β3-tubulin, or total cellular ubiquitinated proteins. As we observed in the HMC3 and GD fibroblast, cathepsin L protein level was increased by SB 412515 in the control iPSC-dopaminergic neurons (Figure 7A). Both intermediate and mature cathepsin L protein levels were increased by SB 412515 in the control iPSC-dopaminergic neurons (Figure 7A). Similar to the observed elevation in GCase protein levels following treatment with the cathepsin L inhibitor in healthy control-dopaminergic neurons (Figure 7A), cathepsin L inhibitor also resulted in a ~1.4-fold increase in in vitro GCase activity in dopaminergic neurons (Figure 7B). Having found that the chemical inhibitor of cathepsin L can increase GCase protein level in the iPSC-dopaminergic neurons, we next asked whether the SB 412515 could correct GCase level in a dopaminergic neuronal PD model of GCase-deficiency. To this end, we used iPSC-derived dopaminergic neurons from a patient with *GBA1*-PD carrying a heterozygous *GBA1*-c.84dupG frameshift mutation, resulting in 50% reduction in GCase level (74). Inhibition of cathepsin L by SB 412515 increased both GCase and LIMP-2 levels in these neurons (Figure 7C), and the levels of pS129-α-synuclein were also significantly reduced by the SB 412515 treatment, while total α-synuclein levels were not changed. The levels of TH, β3-tubulin, lysosomal proton pump subunit ATP6V1D, or total cellular ubiquitinated proteins were not affected by the SB 412515 treatment (Figure 7C). Consistent with the observed elevation in GCase protein levels following treatment with the cathepsin L inhibitor in 84gg *GBA1*-dopaminergic neurons (Figure 7C), we found increased GCase activity upon cathepsin L inhibitor treatment (Figure 7D).

Based on the data from the GD fibroblast cells and dopaminergic neuronal PD model of GCase deficiency, we propose that inhibition of cathepsin L may represent a potential therapeutic strategy for PD and related disorders that exhibit deficiency in lysosomal GCase activity.

## Discussion

Severe loss of GCase activity causes GD (21, 22), and reduced GCase activity increases the risk of PD (5, 8) and DLB (20). Therefore, restoring GCase activity is expected to benefit patients who carry mutations in *GBA1*. In attempts to develop treatments for *GBA1*-associated diseases, several approaches have advanced to clinical trials, including a GCase chaperoning strategy with Ambroxol, which is in phase II clinical trial (NCT05287503), and gene therapy with AAV-WT *GBA1* that is in phase 1/2a (NCT04127578). Since each therapeutic strategy has limitations, new approaches for effective treatment of patients with GCase deficiency are still necessary. Our data suggest a previously unexplored approach, to our knowledge, of increasing lysosomal GCase activity by inhibiting its degradation in the lysosome. We found that inhibition of cathepsin L, a lysosomal cysteine protease, leads to increased levels and activity of GCase by partially reducing its degradation in the lysosome.

Lysosomal cysteine proteases play an important role in intralysosomal protein degradation (44–46). Individual lysosomal cysteine proteases are thought to play redundant and specific functions in cells (44, 45, 75). Here we have shown that cathepsin L is crucial for regulating GCase abundance in the lysosome. Interestingly, although both cathepsin L and cathepsin B are ubiquitously expressed lysosomal cysteine proteases, cathepsin B does not appear to play a significant role in regulating GCase protein levels. Our previous study in cathepsin B–deficient cells revealed no significant change in GCase protein levels (76), suggesting that the regulation of GCase abundance in lysosomes is primarily mediated by cathepsin L. However, our previous study suggested that cathepsin B promotes prosaposin processing to saposin C, an activator of GCase (76). Here, we also found that saposin C levels and the ratio of saposin C/prosaposin were increased in the cathepsin L–KO cells (Figure 1A and Supplemental Figure 1A). While our data suggest that increased GCase activity in cathepsin L–KO cells is primarily due to increased GCase protein levels, it is also possible that increased saposin C levels contribute to the increased activity. It will be of interest to examine the interplay of cathepsins B and L in regulating endogenous lysosomal GCase activity as part of future studies.

Consistent with the data from genetic ablation of cathepsin L, we found that chemical inhibition of cathepsin L increased GCase protein levels, GCase activity in vitro, and GCase activity in living cells. We have further shown that chemical inhibition of cathepsin L in an iPSC dopaminergic neuronal PD model of *GBA1* haploinsufficiency partially corrected reduced GCase protein level and led to reduction of pS129-α-synuclein.

Therefore, targeting cathepsin L–mediated GCase degradation could offer some benefits to *GBA1*-deficient patients. However, previously reported phenotypes of cathepsin L–KO mice, including hair loss, skin thickening, and bone and heart defects, raise concerns about adverse effects of cathepsin L inhibition (77–79). Therefore, it will be important to develop noncovalent and GCase-preferred cathepsin L inhibitors to minimize such adverse effects. Importantly, increasing lysosomal GCase stability using cathepsin L inhibitors could have synergistic effects with other therapeutic strategies, such as chemical chaperoning of GCase, AAV-mediated recombinant GCase delivery, or direct GCase activators. Another potential application of inhibiting GCase degradation would be in enzyme replacement therapy, an effective treatment for type 1 GD. This therapy involves i.v. infusions of recombinant human GCase to correct the GCase deficiency (80). However, the short half-life of the recombinant human GCase enzyme in blood poses a challenge to deliver active recombinant GCase to some target cells and organs, especially the bone (81). We propose that slowing GCase degradation during enzyme replacement therapy could increase the half-life of recombinant GCase and make it more effective. While the feasibility of cathepsin L as a therapeutic target is currently unknown, cathepsin L inhibition has been actively pursued as a potential treatment for COVID-19 (82–84). In this context, cathepsin L mediates cleavage of the S1 subunit of the coronavirus surface spike glycoprotein that is required for host entry and replication (85, 86).

Inhibition of cysteine cathepsins as a potential therapeutic approach has been considered in various disorders, but only a subset of cathepsins has been considered for clinical trials (87). In terms of cathepsin L, as is the case with other cathepsins, it will be important to develop more specific inhibitors to address the substrate overlap among different cathepsin family members.

## Methods

### Antibodies and reagents.

The following antibodies were used in this study: mouse anti–cathepsin L (MilliporeSigma, C4618), mouse anti-GCase (Santa Cruz Biotechnology Inc., sc166407 for immunoblot; Abnova, H00002629-M01 for immunostaining), rabbit anti–LIMP-2 (gift from Michael Schwake), goat anti–cathepsin D (Santa Cruz Biotechnology Inc., sc-6487), mouse anti-LAMP1 (Santa Cruz Biotechnology Inc., sc-20011), mouse anti–α-tubulin (MilliporeSigma, T5168), rabbit anti-prosaposin (Proteintech, 18398-1-AP), mouse anti-ubiquitin (Santa Cruz Biotechnology Inc., sc-8017), rabbit anti-GluCer (Glycobiotech, RAS_0011), mouse anti-ATP6V1D (Santa Cruz Biotechnology Inc., sc-390384), rabbit anti–pS129-α-synuclein (Cell Signaling Technology, 23706), rabbit anti–α-synuclein (Santa Cruz Biotechnology Inc., sc-7011), rabbit anti-TH (MilliporeSigma, 5346S), rabbit anti–b3 tubulin (BioLegend, 802001), and rabbit anti-LC3B (Cell Signaling Technology, 2775). HRP-conjugated second antibodies were obtained from Jackson ImmunoResearch. Fluorescence-labeled secondary antibodies were obtained from Invitrogen. The following reagents were also used in the study: SB 412515, a reversible cathepsin L inhibitor (Cayman Chemicals, 23249), cycloheximide (Cayman Chemicals, 14126), DQ-RED BSA (Thermo Fisher Scientific, D12051), 4-MUG (4-Methylumbelliferyl β-D-glucopyranoside (MilliporeSigma, M3633), PFB-FDGlu (5-[Pentafluorobenzoylamino]Fluorescein Di-β-D-Glucopyranoside) (Invitrogen, P11947), LysoLive Lysosomal β-Glucosidase Assay Kit (MarkerGene, M2775), protease inhibitor (Roche, 05056489001), and Pierce BCA Protein Assay Kit (Thermo Fisher Scientific, 23225). Recombinant GCase (R&D systems, 7410-GH) and recombinant human cathepsin L (R&D systems, 952-CY) were purchased from R&D Systems. Other chemicals were purchased from MilliporeSigma, unless otherwise stated.

### Plasmid, cell culture and transfection.

The CRISPR/Cas9 construct targeting human *CTSL* gene was constructed into pSpCas9(BB)-2A-Puro (PX459) V2.0 (a gift from Feng Zhang, Addgene plasmid, 62988). Target sequences of human *CTSL*-CRISPR/Cas-9 construct are 5′-AGA TGT TCC GGA AAA CTG GG-3′ for the cathepsin L–KO#1, #2, #3 cell line, and 5′-CAG TAT GTT CAG GAT AAT GG-3′ for the cathepsin L–KO#4 cell line. The target sequence of human *GBA1*-CRISP/Cas-9 construct is 5′-GCGACGGATGGAGCTGAGTA-3′. RNAi construct *CTSL*-RNAi #1 (TRCN0000318682), and *CTSL*-RNAi #2 (TRCN0000318611) were purchased from MilliporeSigma. HEK293-FT cells and KO cell lines were maintained in DMEM supplemented with 10% FBS, 1% penicillin/streptomycin. Cells were transfected with Lipofectamine 2000 Transfection Reagent (Invitrogen, 11668-019), according to the manufacture’s instruction. HMC3 cell was purchased from ATCC (catalog CRL3304). HMC3 cells were maintained in EMEM (ATCC) supplemented with 10% FBS and 1% penicillin/treptomycin. Fibroblast cells derived from a from a female patient with Type 3 GD carrying homozygous *GBA1* L444P mutation were obtained from Telethon Network of Genetic Biobank (cell line no. 20516), and fibroblast cells were maintained in DMEM with 15% FBS and 1% penicillin/treptomycin. All cells were cultured in a humidified incubator at 37°C and 5% CO_2_. iPSC cell culture and dopaminergic neuron differentiation were performed as previously described (74). iPSCs (*GBA1*-c.84dupG, C14 clone) derived from skin fibroblasts of a patient with PD carrying a heterozygous *GBA1*-c.84dupG mutation has been previously described (74).

### Human iPSC culture and dopaminergic neuron differentiation.

Detailed methods for iPSC culture and midbrain dopaminergic neuronal differentiation have been described previously (43). Skin fibroblasts of a patient with PD (male) harboring GBA1 heterozygous 84GG mutation were reprogrammed into iPSCs through Northwestern University Stem Cell Core Facility, using Sendai virus–based (SeV-based) delivery of Oct, Sox2, Klf4, and c-Myc. A healthy control iPSC line was purchased from the NINDS Cell Line Repository at the Coriell Institute for Medical Research (ND34769). The iPSCs were maintained in mTeSR1 media (Stemcell Technologies, 85850) and passaged as small chunks every 6–8 days, depending on confluence. All iPSC lines have been tested for mycoplasma on a monthly basis for quality control. Differentiation of iPSCs toward dopaminergic neurons was conducted using a previously established protocol with minor modifications (88). iPSCs were plated as single cells and were treated with small molecules/factors according to the original protocol. At day 13 of differentiation, cells were passaged en bloc (size of 1–2 mm) onto 10 cm culture dishes precoated with poly-d-lysine (PDL) (MilliporeSigma, P1149)/laminin (Invitrogen, 23017-015). At day 25, neurons were passaged onto PDL/laminin-coated culture dishes and were subjected to dopaminergic marker characterization through immunocytochemistry (ICC) using TH (Calbiochem, 657012), FOXA2 (Santa Cruz Biotechnology Inc., sc-101060), LMX1A (MilliporeSigma, 10533) antibodies together with neural-specific marker β-III-tubulin (TUJ1) antibody (BioLegend, 802001 and 801202) at day 30. After day 40, matured neurons were maintained in Neurobasal media (Invitrogen, 21103049) containing Neurocult SM1 (Stemcell Technologies, 5711) without neuralization growth factors.

### Generation of KO cell lines.

HEK293-FT cells grown in 12-well plates were transfected with the CRISPR/Cas9 construct targeting the human *CTSL* gene. One day after transfection, cells were trypsinized and reseeded on a new 10 cm dish with low density (~50 cells in 10 cm dish), and cultured until single cells formed cell colonies. Clonal colonies were picked from the 10 cm culture dish and expanded in new culture dishes. KO clones were identified by immunoblot analysis using cathepsin L antibody.

### Mouse brain lysates, cultured cell lysis, and immunoblotting.

CTSL^–/–^ mice are previously characterized (78). Detailed methods for WT and CTSL^–/–^ mouse brain lysate preparation are previously described (89). Cortical brain lysates from 5-month-old WT and *Ctsl^−/−^* mice were used for immunoblot analysis. HEK293-FT cells were seeded at a density of 100,000–150,000 cells per well in 12-well plates. Two days later, cells were harvested for biochemical assays, unless otherwise stated. Cells were lysed with 2× SDS sample buffer (100 mM Tris-Cl [pH 6.8], 4% [w/v] SDS, 0.05% [w/v] bromophenol blue, 20% [v/v] glycerol, 200 mM dithiothreitol). Cell lysates were subjected to SDS-PAGE. Proteins were then transferred to nitrocellulose membranes using Trans-Blot Turbo Transfer system (Bio-Rad). The membranes were incubated overnight with the indicated primary antibodies. All primary antibodies were diluted in an antibody dilution buffer (25 mM Tris, 0.15M NaCl, 0.05% Tween-20, 5% BSA, 0.05% sodium azide). Primary antibodies were visualized using the appropriate horseradish peroxidase conjugated secondary antibody (anti-mouse (Jackson ImmunoResearch, 115-035-146), anti-rabbit (Jackson ImmunoResearch, 111-035-144), anti-goat (Jackson ImmunoResearch, 805-035-180), and SuperSignal West Femto Maximum Sensitivity Substrate (Thermo Fisher Scientific, 34096). Chemiluminescence signals were taken with the ChemiDoc MP System (Bio-Rad) with a 16-bit CCD camera. Signal accumulation mode was used to take images at progressively longer exposure times. This allowed for acquisition of immunoblot images with band intensities within the linear range of the system. Quantification of protein levels was done using Bio-Rad ImageLab software and ImageJ (NIH) using nonsaturated raw image files.

### In vitro GCase cleavage with recombinant human cathepsin L.

In total, 200 ng of recombinant human GCase was mixed with 150 ng activated recombinant human cathepsin L in 50 µL assay buffer (50 mM MES, 5 mM DTT, 1 mM EDTA, 0.005% [w/v] Brij-35, pH 6.0) for indicated times at room temperature. After the reaction, 50 µL of 4× SDS sample buffer was added into reaction mixtures to stop the reaction. Samples were then analyzed with immunoblot using GCase antibody or cathepsin L antibody.

### Cycloheximide treatment.

Cells were seeded at a density of ~300,000 cells per 35 mm culture dish. Approximately 30 hours later, culture media were replaced with fresh culture medium with 100 µg/mL cycloheximide. Cells were then collected at indicated time points and lysed with RIPA buffer containing protease inhibitor (Roche, 0469311600). Protein concentrations were measured by the BCA protein assay kit. In total, 20 µg of lysates were subjected to immunoblot analysis. GCase levels were normalized with tubulin levels.

### DQ-red BSA assay.

Cells were seeded at a density of ~150,000 cells per 35 mm glass bottom culture dish. Approximately 30 hours later, cells were washed twice with PBS. Cells were then incubated in OPTIM-MEM medium with 10 µg/mL DQ-Red BSA for 4 hours. Cells were then washed with PBS and fixed with 4% formaldehyde in PBS for 15 minutes. After washing with PBS, fluorescent cell images were taken by a widefield epifluorescence microscope system with a red channel filter.

### In vitro GCase activity assay.

Cells were lysed with sonication in ice-cold GCase lysis buffer (0.25% Triton X-100, 0.25% Taurocholic acid, 1 mM EDTA in citrate/phosphate buffer [pH 5.4]). Protein concentrations were measured by BCA method. GCase activity in 10 µg of cleared cell lysates were assayed in 50 mL of GCase assay buffer (1 mM 4-MUG, 0.25% Triton X-100, 1% BSA, 0.25% [w/v] taurocholic acid, 1 mM EDTA, in citrate/phosphate buffer [pH 5.4]). Enzyme reaction mixtures were incubated for 30 minutes at 37°C, and the enzyme reaction was stopped by the addition of equal volumes of 1M glycine, pH 12.5. In endpoint mode, 4-methylumbelliferone (4MU) fluorescence (excitation wavelength [EX], 355 nm; emission wavelength [EM], 460 nm) was measured with SpectraMax i3 (Molecular Devices). The fluorescence value of control no-cell lysates was subtracted from 4MU fluorescence values.

### LysoLive lysosomal GCase assay.

Live-cell lysosomal GCase assay was performed by using LysoLive Lysosomal β*-*Glucosidase Assay Kit (M2775) from MarkerGene. In brief, cells were seeded onto poly-D-lysine–coated 35 mm glass-bottom dishes with a cell density of ~200,000 cells per dish. One day later, cells were washed with PBS and incubated with LysoLive-GlucGreen with 1:2,500 dilution in 2 mL of OPTI-MEM medium (Invitrogen) for about 16 hours. For imaging, cells were washed twice with PBS, prior to the addition of 2 mL of MakerGene-Opti-Klear Live Cell Imaging Buffer. For the experiment testing the effect of SB 412515 on LysoLive GCase activity, approximately 14 hours before imaging, either 7.5 μM SB 412515 or equivalent volume of DMSO was added in the LysoLive-GlucGreen labeling media. Images were taken by a widefield epifluorescence microscope system equipped with bandpass filters with EX/EM: 490/520 nm. The acquisition settings were kept the same for all imaging when fluorescence intensity was compared.

### GCase assay using PFB-FDGlu.

Cells were seeded onto 12-well plate with cell density of approximately 150,000 cells per well. One day later, cells were treated with either DMSO or 7.5 μM SB 412515 for approximately 20 hours. Treated cells were washed with PBS twice and incubated in 1 mL of OPTI-MEM medium (Invitrogen) with 100 μM PFB-FDGlu, a selective lysosomal GCase substrate, for 2 hours. Cells were then washed twice with PBS and lysed with 300 µL of RIPA buffer (Pierce). In endpoint mode, fluorescence of 100 µL of cell lysates (EX/EM: 485/530 nm) was measured with SpectraMax i3 (Molecular Devices). Data were normalized with protein concentration.

### MS-based lipid analysis.

HEK293-FT cells (WT and *CTSL* KO#1) were grown on a 10 cm. Roughly 5 million cells from each 10 cm dish were pelleted with spin at 300*g* for 5 minutes and frozen at –80°C. L444P fibroblast cells were grown on a 15 cm dishes. *GBA1* L444P fibroblast cells were treated with either DMSO or 5 μM SB 412515 for about 20 hours. Roughly 3 million cells from each 15cm dish were pelleted with spin at 300*g* for 5 minutes and frozen at –80°C. At the University of South Carolina Medical Lipidomics Center, lipids were extracted from frozen cell pellets as a service provided by their core facilities. Levels of GluCer were measured by a high-performance liquid chromatography/tandem MS (LC-MS/MS) method. Lipid analysis results were normalized with inorganic phosphate (Pi).

### Immunostaining and imaging.

For the immunostaining of GCase or LIMP-2, cells were fixed with Bouin’s solution for 20 minutes at room temperature. After washing with PBS 3 times, cells were incubated in GDB buffer (30 mM phosphate buffer [pH 7.4], containing 1% goat serum, 0.1% gelatin, 0.3% Triton X-100, and 0.45M NaCl) for 30 minutes. Cells were then incubated in GDB buffer containing GCase or LIMP-2 antibodies overnight at 4°C. Primary antibodies were visualized using goat Alexa Fluor–conjugated secondary antibodies against mouse or rabbit. For the GluCer immunostaining, cells were fixed with 4% formaldehyde in PBS for 15 minutes. After washing with PBS, fixed cells were incubated in Tween-20 blocking solution (PBS containing 0.5 % BSA and 0.01% Tween-20 in PBS) for 30 minutes. Cells were then incubated in Tween-20 blocking solution containing rabbit anti-GluCer antibody (Glycobiotech, RAS_0011) for 45 minutes. Primary antibodies were visualized using goat Alexa Fluor–conjugated secondary antibodies against rabbit. Hoechst 33342 was used for staining the nuclei of fixed cells. Immunostaining images were acquired with a Leica confocal microscope with a 63× oil objective. The confocal microscope settings were kept the same for all scans when fluorescence intensity was compared. Fluorescence intensity measurements were performed using ImageJ (NIH) using nonsaturated raw image files. Data were expressed relative to WT levels as indicated. Pearson’s correlation coefficient of GCase and LIMP-2 were analyzed using Coloc2 plugin (Fiji).

### Statistics.

All values in figures and text refer to mean ± SEM unless otherwise stated. Statistics and graphing were performed using Prism (GraphPad) software. Statistical analysis of data was performed with 2-tailed unpaired *t* test unless otherwise indicated.

### Data availability.

Values for all data points found in graphs are in the Supporting Data Values file.

## Author contributions

MJK and DK conceived and designed the experiments. MJK and SK performed the experiments. TR provided brain lysates from WT and CTSL-KO mice. MJK analyzed the data. MJK and DK wrote the manuscript. All authors read and approved the final manuscript.

## Supplementary Material

Supplemental data

Supporting data values

## Version 1 02/08/2024

Electronic publication

## Version 2 02/20/2024

A typographical error was fixed in the article's title
