# Supplementary material for: Inhibition of cysteine protease cathepsin Lincreases the level and activity of lysosomal glucocerebrosidase
Source: JCI Insight. 2024 Feb 8;9(3):e169594. doi: 10.1172/jci.insight.169594 (PMC10967467; doi:10.1172/jci.insight.169594)
Supplement: Supplemental data [file jciinsight-9-169594-s226.pdf]

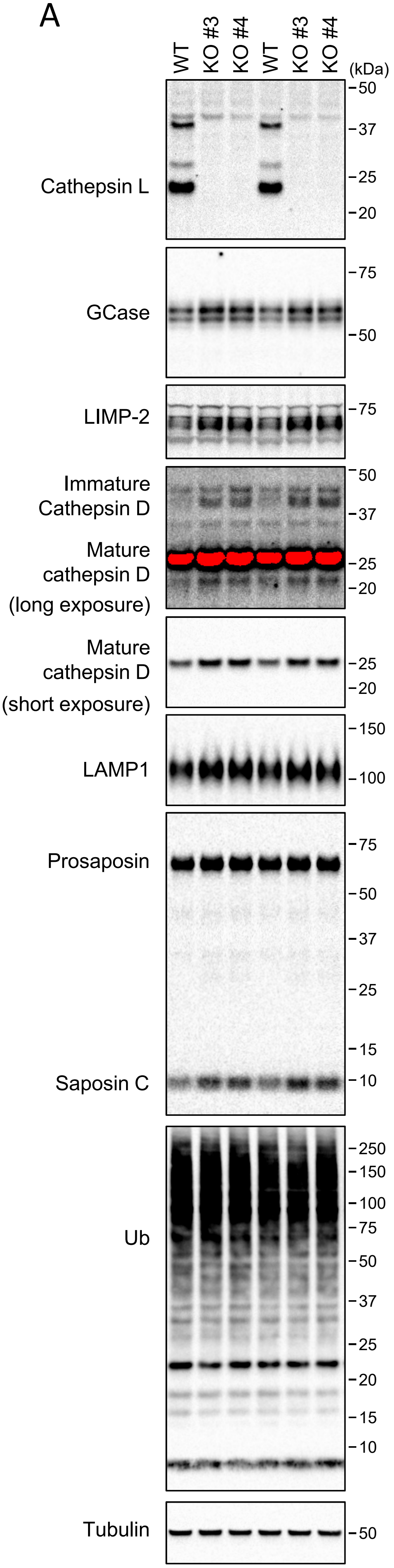

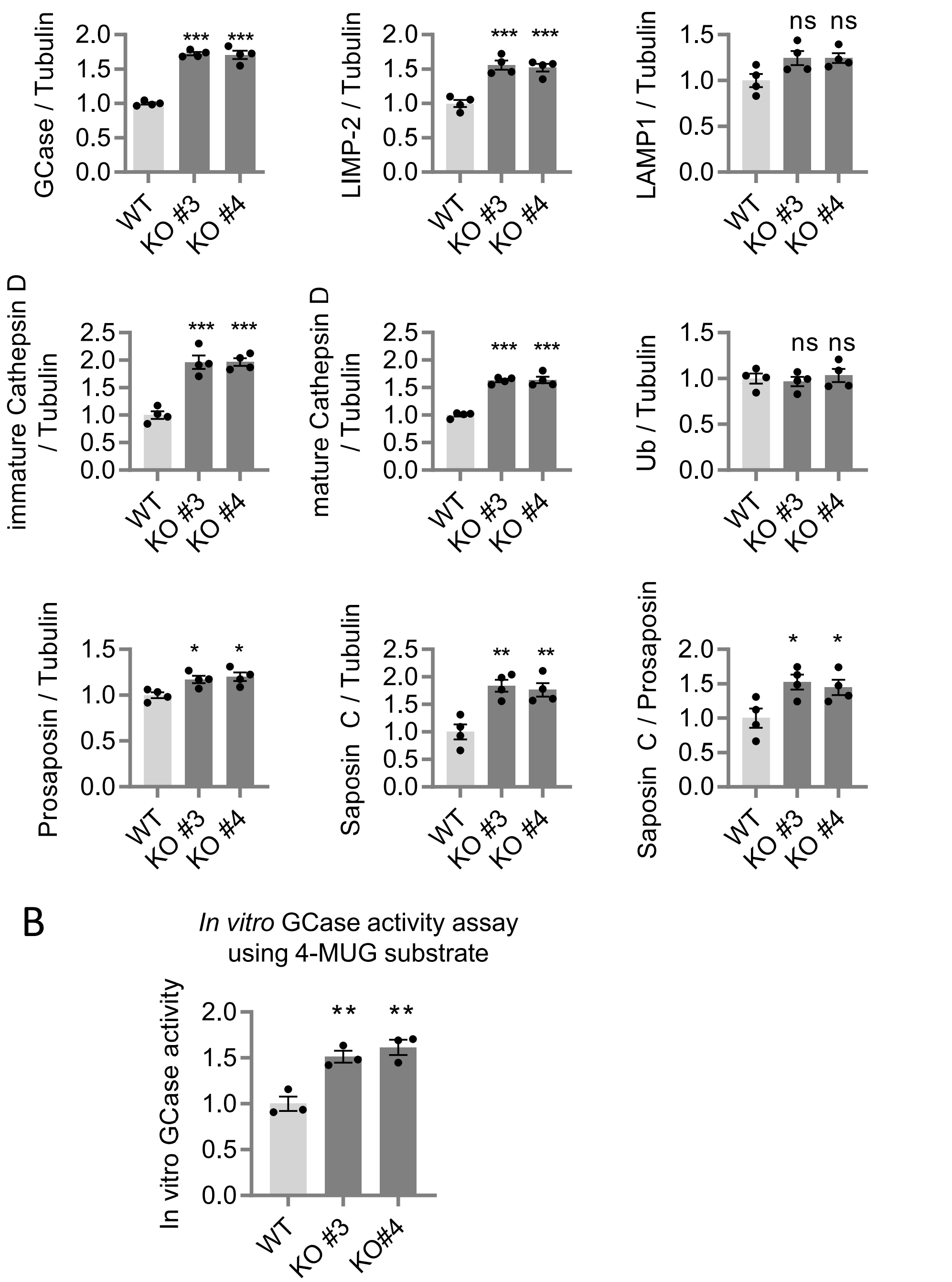

Supplementary Figure 1: GCase protein levels and *in vitro* GCase activities are increased in cathepsin L KO cells (KO #3 and KO #4).

(**A**) Representative immunoblot data and quantification of indicated proteins in WT and two additional cathepsin L KO cell lines (KO #3 and KO #4). KO #3 and KO #4 Cathepsin L KO cell lines were generated with Cas9/CRISPR. Target sequences of human *CTSL*-CRISPR/Cas-9 construct are 5'- AGA TGT TCC GGA AAA CTG GG-3' for the cathepsin L KO #3 cell line, and 5'-CAG TAT GTT CAG GAT AAT GG-3' for the cathepsin L KO #4 cell line. Band intensities were normalized to tubulin, and compared to the WT cells. Data are means ± SEM. One-way analysis of variance (ANOVA) followed by Dunnett's test. n=4, ***p<0.001, **p<0.01, *p<0.05, ns, not significant.

(**B**)) *In vitro* GCase activity assay of WT HEK293-FT and cathepsin L-KO cell lysates. Lysates from WT or cathepsin L-KO cells (KO #3 and KO #4) were assayed for *in vitro* GCase assay using 4-MUG substrates. Data are means ± SEM. One-way analysis of variance (ANOVA) followed by Dunnett's test, **p<0.01, n=3.

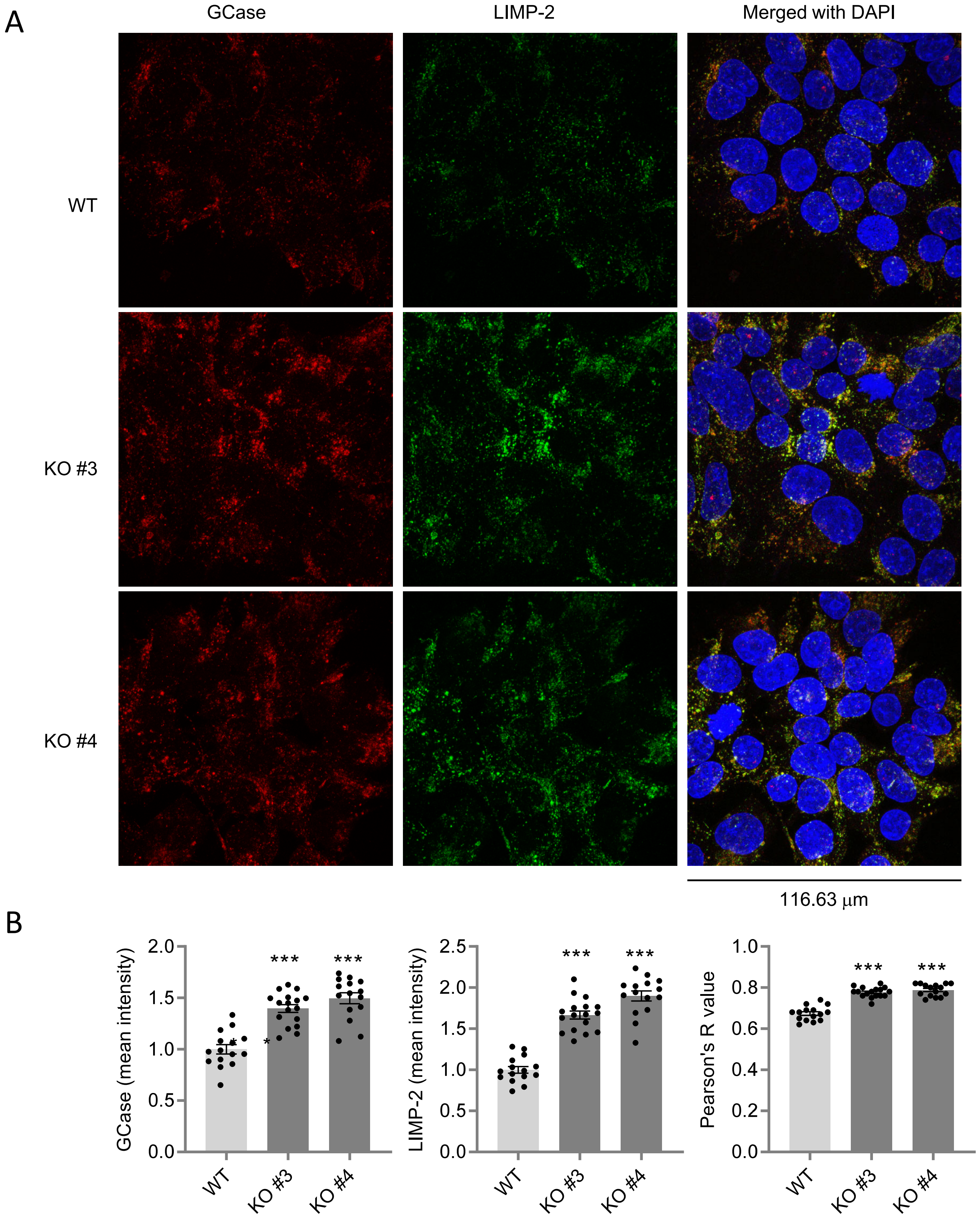

Supplementary Figure 2: Immunofluorescent image of GCase and LIMP-2 in WT and cathepsin L KO cells (KO #3 and KO #4)

(A) Representative immunofluorescent image of GCase and LIMP-2 in WT and cathepsin L KO cells (KO #3 and KO #4). (B) Quantitation of mean fluorescence intensity and Pearson's correlation coefficient of GCase and LIMP-2 are shown. n=15 microscopic fields from two coverslips for WT, 17 microscopic fields from two coverslips for KO #3 and 15 microscopic fields from two coverslips for KO #4 cells. Data are means ± SEM. One-way analysis of variance (ANOVA) followed by Dunnett's test, ***p<0.001.

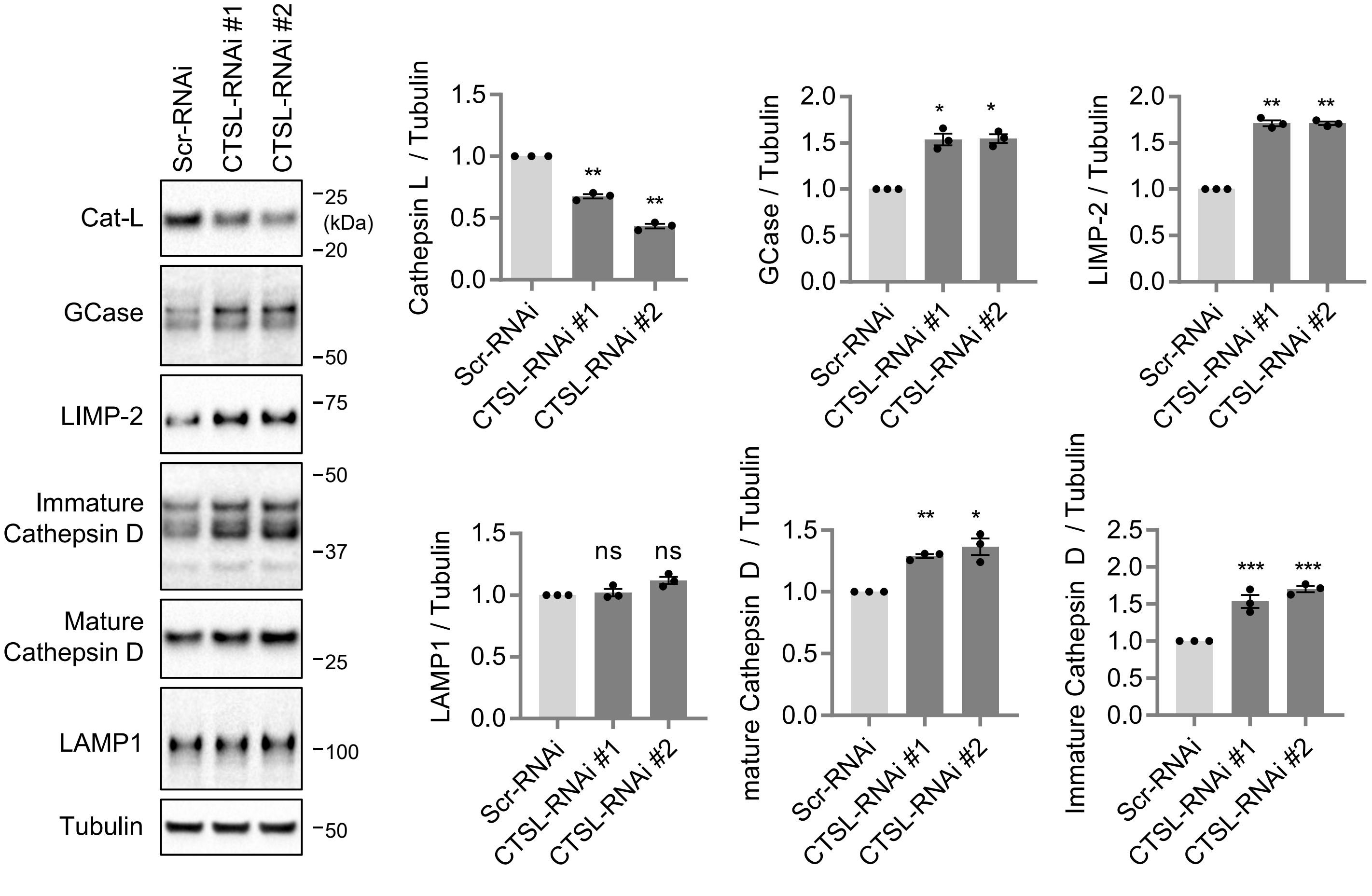

Supplementary Figure 3: Effects of cathepsin L RNAi-knockdown on GCase and LIMP-2 levels in HEK293-FT cells.

HEK293-FT cells were transfected with indicated RNAi constructs. Two days later, transfected cells were analyzed with immunoblotting. Immunoblot band intensities were normalized to tubulin, and compared to Scr-RNAi. Data represent mean ± SEM; *n* = 3, One-way analysis of variance (ANOVA) followed by Dunnett's test. ***p<0.001, **p<0.01, *p<0.05, ns, not significant.

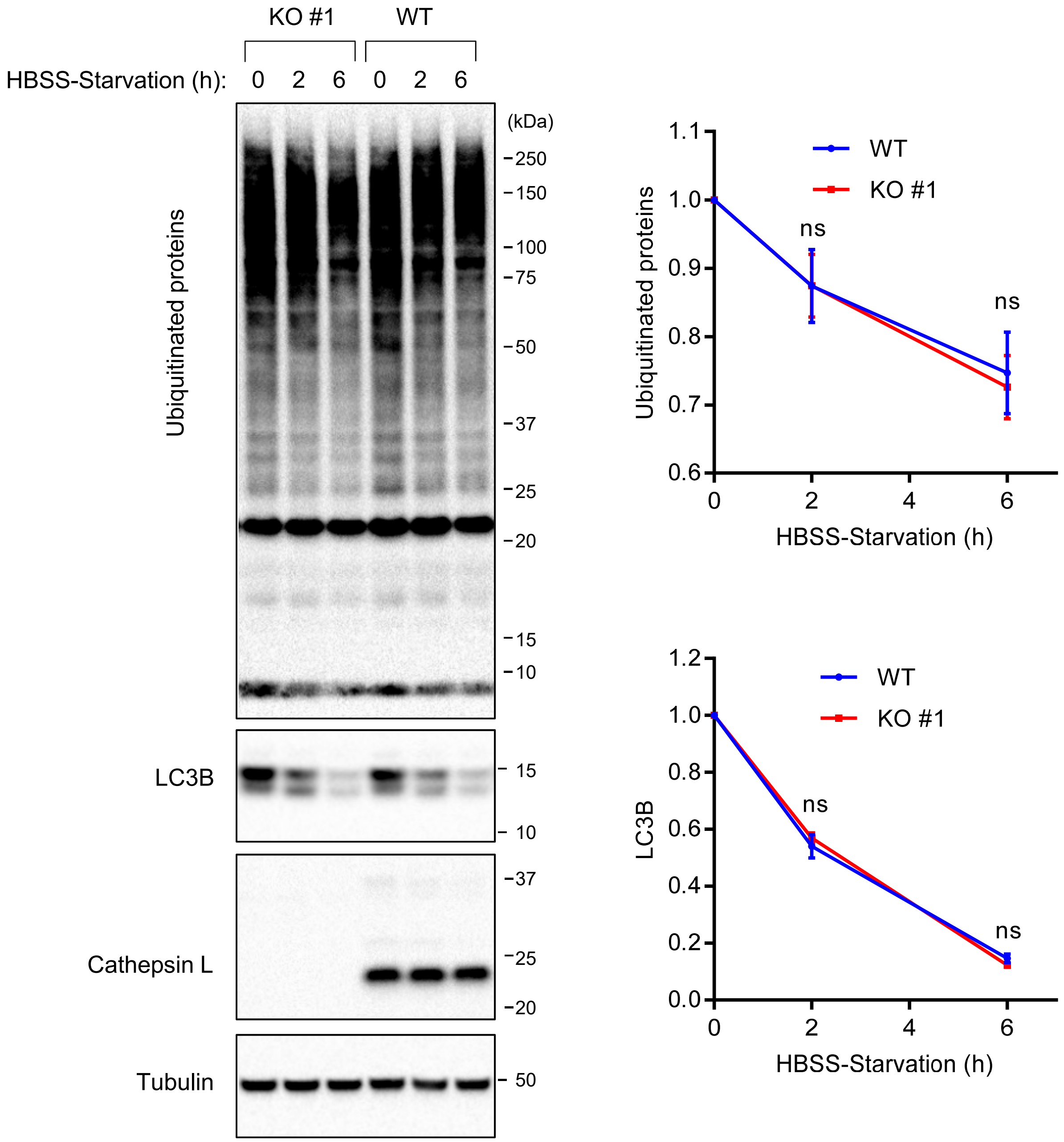

Supplementary Figure 4: Starvation-induced degradative autophagic process in cathepsin L-deficient cells. Representative immunoblot blot from cell lysates from wild-type and cathepsin L-deficient cells (KO #1) starved for indicated times with Hanks' balanced salt solution. Cell lysates were immunoblotted with antibodies against ubiquitin, cathepsin L, LC3B and tubulin. Band intensities were normalized with tubulin and compared with 0-h samples. Data represent mean ± SEM. n=4 independent experiments. Paired two-tailed Student's *t*-test. ns, not significant.

A

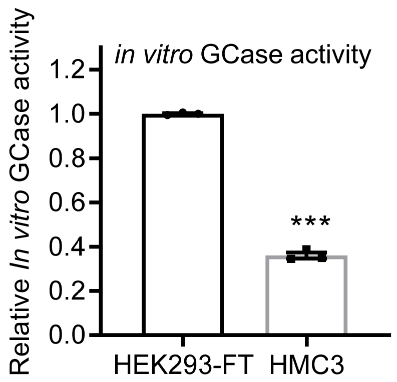

B

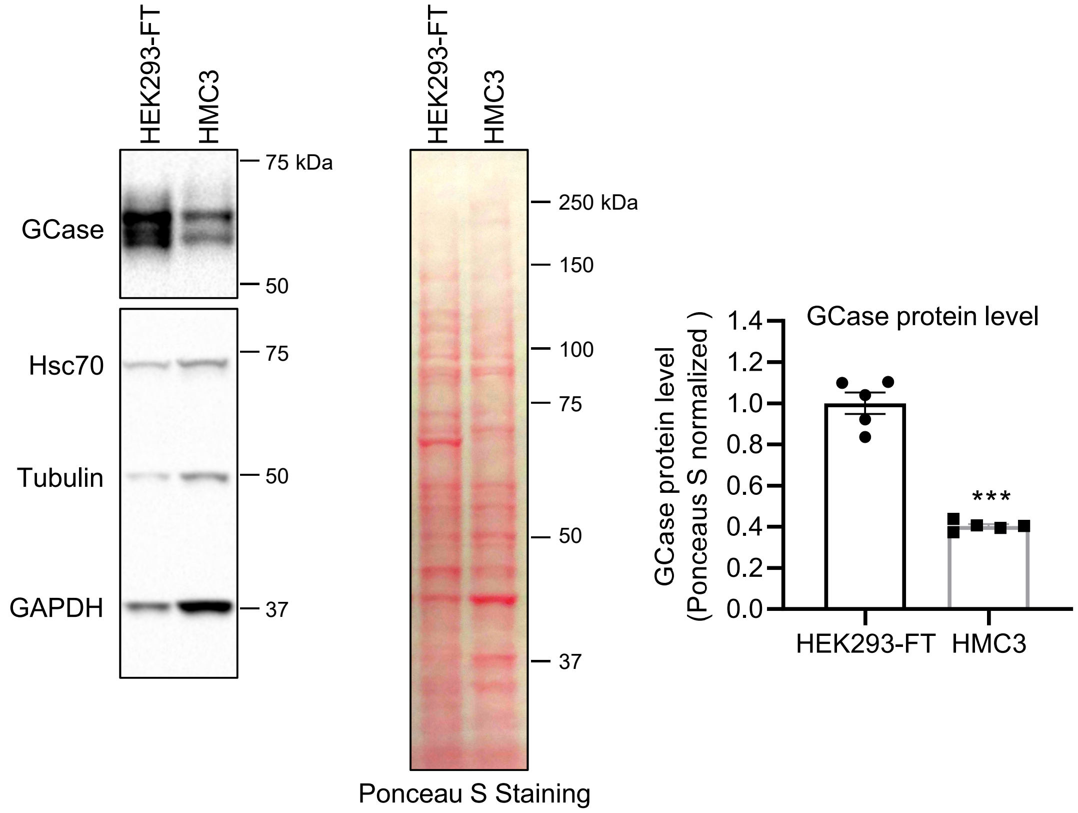

Supplementary Figure 5: Comparison of *in vitro* GCase activity and GCase protein level in HEK293-FT and HMC3 cells

(A) *In vitro* GCase activities in HEK293-FT and HMC3 cell lysates. 10 µg of lysates from HEK293-FT or HMC3 cells were assayed for *in vitro* GCase assay using 4-MUG substrates. Data are means ± SEM. Two-tailed unpaired t-test. ***$p<0.001$, n=3. (B) Analysis of GCase protein levels in HEK293-FT and HMC3 cells. Western blot analysis of 5 µg of cell lysates from HEK293-FT and HMC3 cells with indicated antibodies. The GCase protein band intensities were normalized to Ponceau S signal and then compared to the HEK293-FT cells. Data presented as mean ± SEM. Tw-tailed unpaired t-test ***$p<0.001$, n=5.

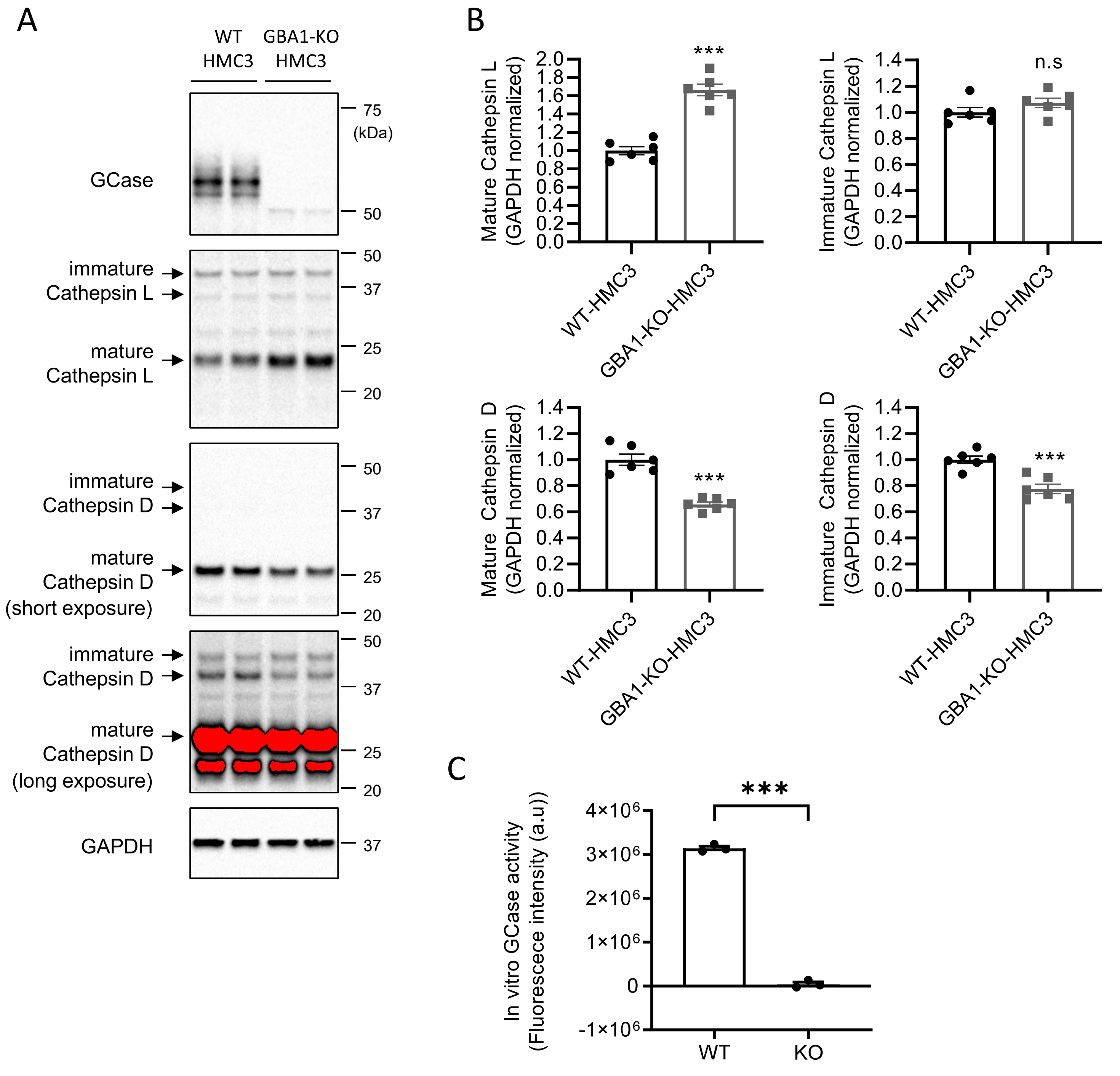

Supplementary Figure 6: HMC3 cells lacking GCase show increased levels of cathepsin L and decreased levels of cathepsin D

(A) Representative immunoblot data showing the levels of cathepsin L and cathepsin D in GBA1-deficient HMC3 cells and wild-type HMC3 cells. *GBA1*-knockout HMC3 cell was generated by Cas9/CRISPR. The target sequence of human *GBA1*-CRISP/Cas-9 construct is 5′-GCGACGGATGGAGCTGAGTA-3′. Cell lysates from six independent cell culture wells were used for both wild-type (WT) and *GBA1*-knockout HMC3 cells (*GBA1*-KO-HMC3). Saturated pixels are shown in red color.(B) The protein band intensities were normalized to GAPDH and then compared to the wild-type HMC3 cells. Saturated pixels are shown in red color. Statistical analysis using a two-tailed unpaired t-test were used. ***p<0.001, "n.s" denotes non-significance. (C) *In vitro* GCase activity assay of WT HMC3 and GBA1-KO HMC3 cell lysates. Lysates from WT or GBA1-KO cells were assayed for *in vitro* GCase assay using 4-MUG substrates. Data are means ± SEM. Two-tailed unpaired t-test, ***p<0.001, n=3.

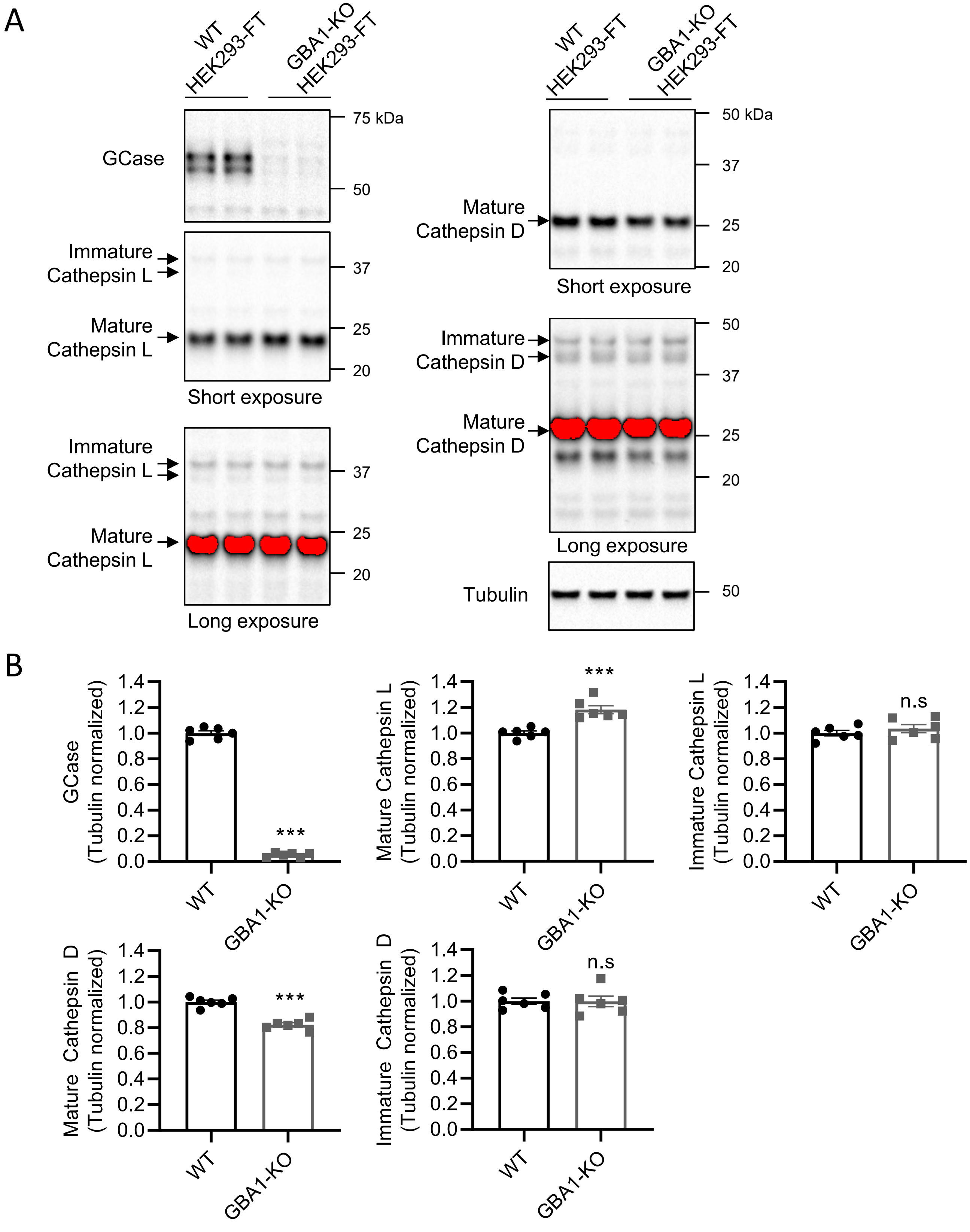

Supplementary Figure 7: *GBA1*-KO HEK293-FT cells exhibit elevated levels of mature cathepsin L and reduced levels of mature cathepsin D

(A) Representative immunoblot data showing the levels of cathepsin L and cathepsin D in GBA1-deficient HEK293-FT cells and wild-type HEK293-FT cells. Cell lysates from six independent cell culture wells were used for both wild-type (WT) and GBA1-KO HEK293-FT cells. Saturated pixels are shown in red color. (B) The protein band intensities were normalized to tubulin and then compared to the wild-type. Statistical analysis using a two-tailed unpaired t-test were used. ***p<0.001, "n.s" denotes non-significance.

.

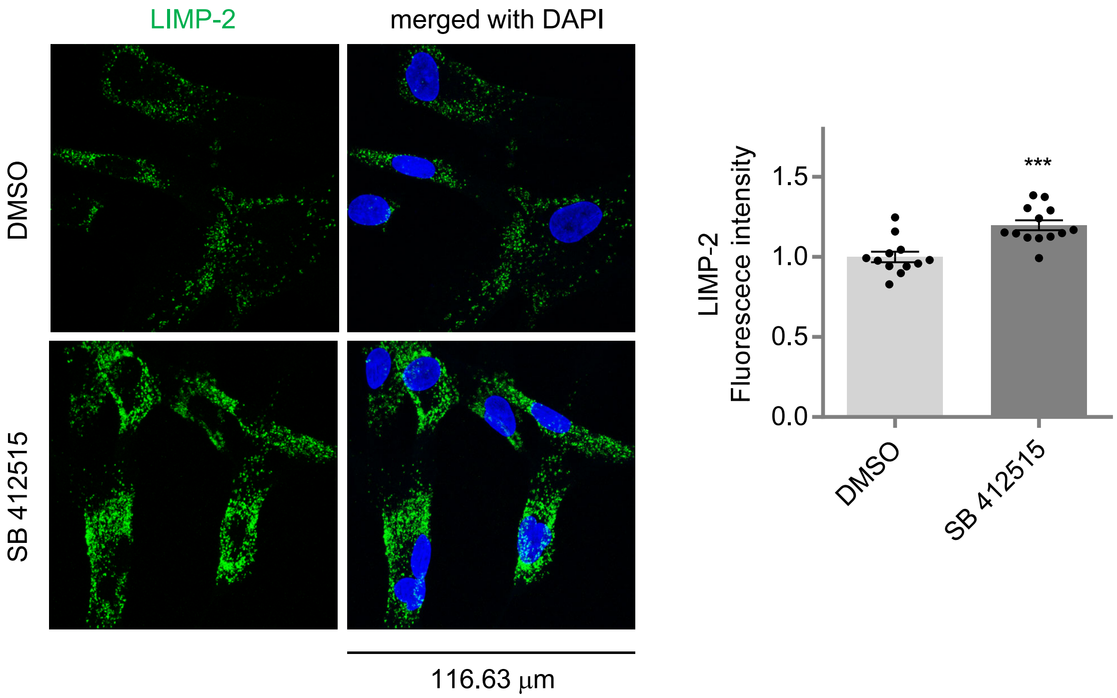

Supplementary Figure 8: Effects of SB 412515, a reversible cathepsin L inhibitor, on LIMP-2 levels in GD-fibroblast cells.

Representative immunofluorescent image of LIMP-2 in GD-fibroblast cells. GD-fibroblast cells were treated with either DMSO or 5 μM SB 412515 for ~20 hrs. Quantitation of mean fluorescence intensity of LIMP-2 is shown. n=12 microscopic fields from two coverslips for DMSO-treated cells, n=13 microscopic fields from two coverslips for SB 412515-treated cells, ***p<0.001, two-tailed unpaired t-test.
